# Dietary inflammatory index and its association with risk of metabolic syndrome and its components: a systematic review and Meta-analysis of Observational studies

**DOI:** 10.1186/s41043-024-00580-w

**Published:** 2024-06-19

**Authors:** Farnush Bakhshimoghaddam, Rezvan Chaharlang, Anahita Mansoori, Narges Dehghanseresht

**Affiliations:** 1 Student Research Committee, Ahvaz Jundishapur University of Medical Sciences, Ahvaz, Iran; 2 Nutrition and Metabolic Diseases Research Center, Clinical Sciences Research Institute, Ahvaz Jundishapur University of Medical Sciences, Ahvaz, Iran; 3 Department of Nutrition, School of Allied Medical Sciences, Ahvaz Jundishapur University of Medical Sciences, Ahvaz, Iran

**Keywords:** Inflammation, Dietary inflammatory index, Metabolic syndrome, Obesity, Meta-analysis

## Abstract

**Background:**

It is believed that the progression and development of metabolic syndrome (MetS) are associated with low-grade systemic inflammation. Several studies have suggested that the Dietary Inflammatory Index (DII), which estimates the inflammatory potential of diets, is associated with MetS. This systematic review and meta-analysis aimed to determine the relationships between DII and the MetS and its components.

**Methods:**

Relevant articles published in English from inception to May 2024 were identified by searching electronic databases including PubMed, Scopus, and Web of Science. We included studies that reported the odds ratio (OR), relative risk (RR), or hazard ratio for the association of DII with the MetS and its components. Effect sizes were pooled using a random effects model.

**Results:**

A total of three prospective studies and 22 cross-sectional studies were included in this meta-analysis. The results showed that pro-inflammatory diets were significantly associated with an increased risk of MetS in cohort studies (RR: 1.33; 95% confidence interval [CI]: 1.19–1.48) and cross-sectional studies (OR:1.24; 95% CI: 1.11–1.38). Meta-analysis of cross-sectional studies showed that a higher DII score was significantly associated with higher odds of hypertension (OR = 1.19; 95% CI = 1.10–1.28) and hyperglycemia (OR = 1.18; CI = 1.06–1.32). The pooled OR comparing the highest versus lowest category of DII with the odds of abdominal obesity and hypertriglyceridemia was significant only after adjustment for covariates.

**Conclusions:**

In general, higher DII is associated with a higher risk of MetS and some of its components. Based on the findings, dietary interventions should be considered for preventing MetS from the inflammatory perspective.

**Supplementary Information:**

The online version contains supplementary material available at 10.1186/s41043-024-00580-w.

## Highlights

Metabolic Syndrome (MetS), is a major risk factor for cardiovascular disease, type 2 diabetes mellitus, and cancer.

Low-grade systemic inflammation is associated with the progression and development of MetS.

The Dietary Inflammatory Index (DII), a measure of the inflammatory potential of diets, is associated with MetS.

A high DII score is expected to be associated with a higher risk of chronic diseases, including MetS.

**Supplementary Information:**

The online version contains supplementary material available at 10.1186/s41043-024-00580-w.

## Introduction

Metabolic syndrome (MetS) is a cluster of metabolic risk factors that contribute to the development of type 2 diabetes (T2DM) and cardiovascular disease (CVD), as well as impose significant economic burdens on the healthcare system [1]. According to the consensus definition of the International Diabetes Federation, the American Heart Association, and the National Heart, Lung, and Blood Institute, risk factors include elevated blood pressure, dysglycemia, dyslipidemia, and central obesity [2]. Recent data published in 2022 reported that the global prevalence of MetS ranges from 12.5 to 31.4%, depending on region and population, with an upward trend in most cases [3]. Several mechanisms and factors contribute to MetS development, such as genetics, dysfunction of adipose tissue, insulin resistance, oxidative stress, and chronic inflammation [4]. As a result of chronic inflammation several pro-inflammatory cytokines are secreted, such as adiponectin, leptin, tumor necrosis factor-alpha (TNF-a), interleukin-1 (IL-1), and IL-6 [5]. Chronic inflammation can be triggered by pro-inflammatory diets and cell death that causes inflammation locally [6].

In recent years, evidence has suggested that lifestyle factors, particularly dietary habits, play an essential role in MetS development or prevention [7]. Mediterranean and Dietary approaches to stop hypertension (DASH) diets, which are rich in vegetables, fruits, whole grains, nuts, legumes, olive oil, and fish, reduce C-reactive protein (CRP) and systemic inflammation compared to unhealthy diets [8, 9]. A Western diet (rich in saturated fats, refined grains, simple carbohydrates, processed foods and meat, and sodium) combined with a sedentary lifestyle can induce a state of chronic metabolic inflammation, which contributes to the development of many common metabolic disorders [6]. In this context, the Dietary Inflammatory Index (DII), the scoring algorithm of 45 food parameters, was developed and validated to measure the individual’s inflammatory potential comprehensively [10]. In summary, lower DII scores indicate a less inflammatory diet, while higher DII scores indicate a more inflammatory diet.

In several systematic reviews, associations have been investigated between DII and health outcomes, such as cancer [11], CVD risk [12], pregnancy outcomes [13], and mental health [14]. Although only two systematic reviews and meta-analyses in 2018 and 2021 have investigated the association between dietary inflammatory potential and MetS, the results were conflicting [15, 16]. Thus, the association between dietary inflammatory potential and MetS and its components emphasizes the necessity of a comprehensive systematic review of this issue. Moreover, the number of papers on this topic has increased significantly since 2021.

Therefore, we conducted systematic reviews and meta-analyses of observational studies that assessed the association between DII and MetS and its components, such as abdominal obesity, low HDL cholesterol, hypertriglyceridemia, hyperglycemia, and hypertension.

## Materials and methods

This systematic review and meta-analysis follow the principles of the Preferred Reporting Items for Systematic Reviews and Meta-Analyses (PRISMA) statement guidelines [17]. The study aimed to assess the association between DII and MetS and its components. The protocol was registered in the International Prospective Register of Systematic Reviews (PROSPERO) database (CRD42023449592).

### Search strategy

The Web of Science, Scopus, and PubMed databases were searched from inception to May 2024. Experts in this field have developed the MeSH and non-MESH terms, which included the following terms: (inflammatory diet* OR dietary inflammatory index OR anti-inflammatory diet* OR dietary score* OR inflammatory potential intake OR pro-inflammatory diet* OR dietary inflammatory potential score OR dietary inflammatory score OR DII) AND (Metabolic syndrome* OR insulin resistance syndrome OR insulin resistant syndrome OR syndrome x OR x syndrome OR metabolic cardiovascular syndrome OR cardio-metabolic syndrome OR glucose metabolism disorders OR MetS OR MetSyn OR ((Hypertension or HP or high blood pressure) and (Hyperlipidemia or lipid disorder)) OR ((Hypertension or HP or high blood pressure) and (hyperglycemia or diabetes or T2DM)) OR ((Hypertension or HP or high blood pressure) and (obesity or overweight)) OR ((Hyperlipidemia or lipid disorder) and (hyperglycemia or diabetes or T2DM)) OR ((Hyperlipidemia or lipid disorder) and (obesity or overweight)) OR ((hyperglycemia or diabetes or T2DM) and (obesity or overweight))) (Supplemental Table 1). No geographic restrictions or language were imposed. Manual searches of cited reference lists were also conducted to identify articles that may not have been found in electronic databases.

### Eligibility criteria

This meta-analysis included studies with the following conditions: (a) performed on individuals over 18 years of age; (b) cohort and cross-sectional studies; and (c) studies that reported odd ratios (OR), hazard ratios (HRs), or risk ratios (RRs), with 95% confidence interval (CI). Studies were excluded if they were: (a) review articles, systematic reviews, meta-analyses, conference proceedings, book chapters, patents, case reports, and editorials/letters; and (b) studies on children or adolescents.

### Data extraction

Two independent reviewers (ND and RCH) screened the titles and abstracts of relevant studies and performed the study selection, whereas a chief investigator (FB) was present to resolve any disagreements. Studies relevant to inclusion were identified by reviewing the full text of the potentially eligible articles. From each study, the following data was recorded: First author, year of publication, country, WHO region (American, European, African, Eastern Mediterranean, Western Pacific, and Southeast Asian), cohort study period, MetS definitions, effect estimates (OR, HR, or RR), comparison level and multivariable analyses adjusting for covariates. We extracted the OR, RR, or HR values with the most adjustments.

### Quality assessment

The quality of cross-sectional and cohort studies was assessed independently by two reviewers (FB and ND) using the Risk Of Bias In Non-randomised Studies-of Interventions (ROBINS-I) tool [18]. Briefly, the tool incorporates seven domains: (1) bias due to confounding, (2) bias in the selection of study participants, (3) bias in the measurement of exposure, (4) bias due to misclassification of exposure during follow-up, (5) bias due to missing data, (6) bias in the measurement of outcomes, and (7) bias in the selection of reported outcomes. The risk of bias was then defined based on three groups: low (low risk of bias in all domains), moderate (low or moderate risk of bias in all domains), and high (serious risk of bias in at least one domain).

### Statistical analysis

The meta-analysis was performed separately for cross-sectional and cohort studies. We used the ORs reported in the studies included in the meta-analysis for the meta-analysis of cross-sectional studies. For the cohorts, we converted the reported HRs and ORs to RRs and performed the meta-analysis based on the calculated RRs. First, we calculated Ln OR (and 95% CI) to normalize the distribution and calculate the summary effect size (ES). A random-effects model [DerSimonian and Laird method [19]] was used to calculate the summary ES for the comparison of the highest versus lowest category of DII and the risk of metabolic syndrome and its components (abdominal obesity, high blood pressure, hyperglycemia, hypertriglyceridemia, and low HDL-cholesterol). To assess heterogeneity, the Q test and I^2^ statistic were calculated. The Q test was considered significant when the p-value was below 0.05. I^2^ values of more than 50% were considered a high degree of heterogeneity between studies. To detect the possible source of heterogeneity, subgroup analysis was performed based on study design (cross-sectional vs. cohort), WHO region [Eastern Mediterranean Region (EMR), Americas Region (AMR), European Region (EUR), South-East Asia Region (SEAR), Western Pacific Region (WPR)], diagnostic criteria for MetS [The Third Report of the National Cholesterol Education Program Expert Panel on Detection, Evaluation, and Treatment of High Blood Cholesterol in Adults (NCEP ATP III), Joint Interim Statement (JIS), The International Diabetes Federation Criteria (IDF), and other], and adjustments of possible confounding variables (BMI, physical activity, and energy intake). For the dose-response meta-analysis, studies with at least three exposure categories were included. The total number of participants, cases, mean DII, and odds ratio with 95% CI for each DII category of the included studies were extracted. Where the median or mean of the DII was not reported, the midpoint of the lower and upper limits was used as an approximation. If the highest or lowest category was open-ended, we assumed that the category had the same range interval as the adjacent category. Potential non-linear relationships between DII and the odds of MetS and its components were assessed using restricted cubic splines, and generalized least squares trend estimation was used to measure linear dose-response relationships. Publication bias was evaluated by visual examination of the funnel plot and statistical test of funnel plot asymmetry using Egger’s test. In addition, sensitivity analyses were performed to specify if a particular study affected the outcomes.

## Results

### Description of studies

We identified 1033 references from databases and manual searches published from inception to 2024. The remaining 565 articles were screened using the title and abstract after removing duplicate articles (*n* = 468). A total of 36 articles were found to be eligible for full-text review after full screening based on the inclusion criteria. Finally, 25 eligible cohort and cross-sectional studies were identified and qualified for the final meta-analysis.

Supplemental Table 2 shows the characteristics of the articles included. Of the 25 articles in the body of evidence, 22 were cross-sectional [20–41], and three were prospective cohort studies [42–44]. In this study, countries were classified based on WHO regions. A total of five studies originated from the AMR [26, 31, 36, 40, 43], six from the EUR [22, 27, 34, 39, 41, 44], six from the WPR [20, 21, 23–25, 33], six from the EMR [28–30, 32, 35, 38], and two from the SEAR [37, 42]. Participants number ranged from 100 to 157,812, with an average age between 18 and 79. Most studies reported both genders in combination, though one study assessed only women [26]. A majority of the included articles used NCEP-ATP III criteria (*n* = 15) [21, 24, 25, 28–30, 32, 34–37, 40–43] for defining MetS. For DII calculating, most studies utilized validated FFQs (*n* = 20) [20, 22–32, 34, 35, 38–43], whereas the remaining studies used dietary recalls (*n* = 3) [21, 33, 37], food records (*n* = 1) [44], and not specified (*n* = 1) [36]. According to ROBINS-I, three studies were assessed as having a low risk of bias [42–44], and 22 studies were assessed as having a moderate risk of bias [20–41]. (Supplemental Table 3).

### Findings from the systematic review

A total of three cohort studies found positive associations between DII and MetS risk [42–44]. In the Korean Genome and Epidemiological Studies Health Examination cohort, which included 157,812 participants with a mean follow-up of 7.4 years, higher DII scores were associated with an increased risk of MetS (HR: 1.31; 95% CI: 1.15–1.49; P trend = 0.002) and its five components [42]. Another cohort study of 399 participants found a positive association between those with the higher DII and the incidence of MetS (HR: 1.99; 95% CI: 1.03, 3.85; P trend = 0.04) and its components, except HDL-c and FBS, over 13 years of follow-up [43]. Similarly, in the Supplémentation en Vitamines et Minéraux AntioXydants cohort with 3726 participants and 13 years of follow-up, Neufcourt et al. [44] found that a diet with a high DII score was significantly associated with a higher risk of MetS (OR: 1.39, 95% CI: 1.01–1.92, *P* = 0.047) and its components, such as higher blood pressure and triglycerides, and with lower HDL-c levels (Supplemental Table 2).

Overall, 13 cross-sectional studies reported a significant association between DII and MetS [21, 23–27, 29, 30, 32, 34–37]. Mazidi et al. [36] used data from 17,689 participants selected from the US National Health and Nutrition Examination Survey and suggested that the metabolic syndrome and its components were associated with a higher DII score. In addition, a statistically significant association between MetS and DII was found in the Fasa cohort study of 10,017 participants [29]. However, in some cross-sectional studies, there was a null association between the DII score and MetS [20, 22, 28, 31, 33, 38–41] (Supplemental Table 2).

### Findings from the meta-analysis

#### Dietary inflammatory index and metabolic syndrome and its components

**Fig. 1:**
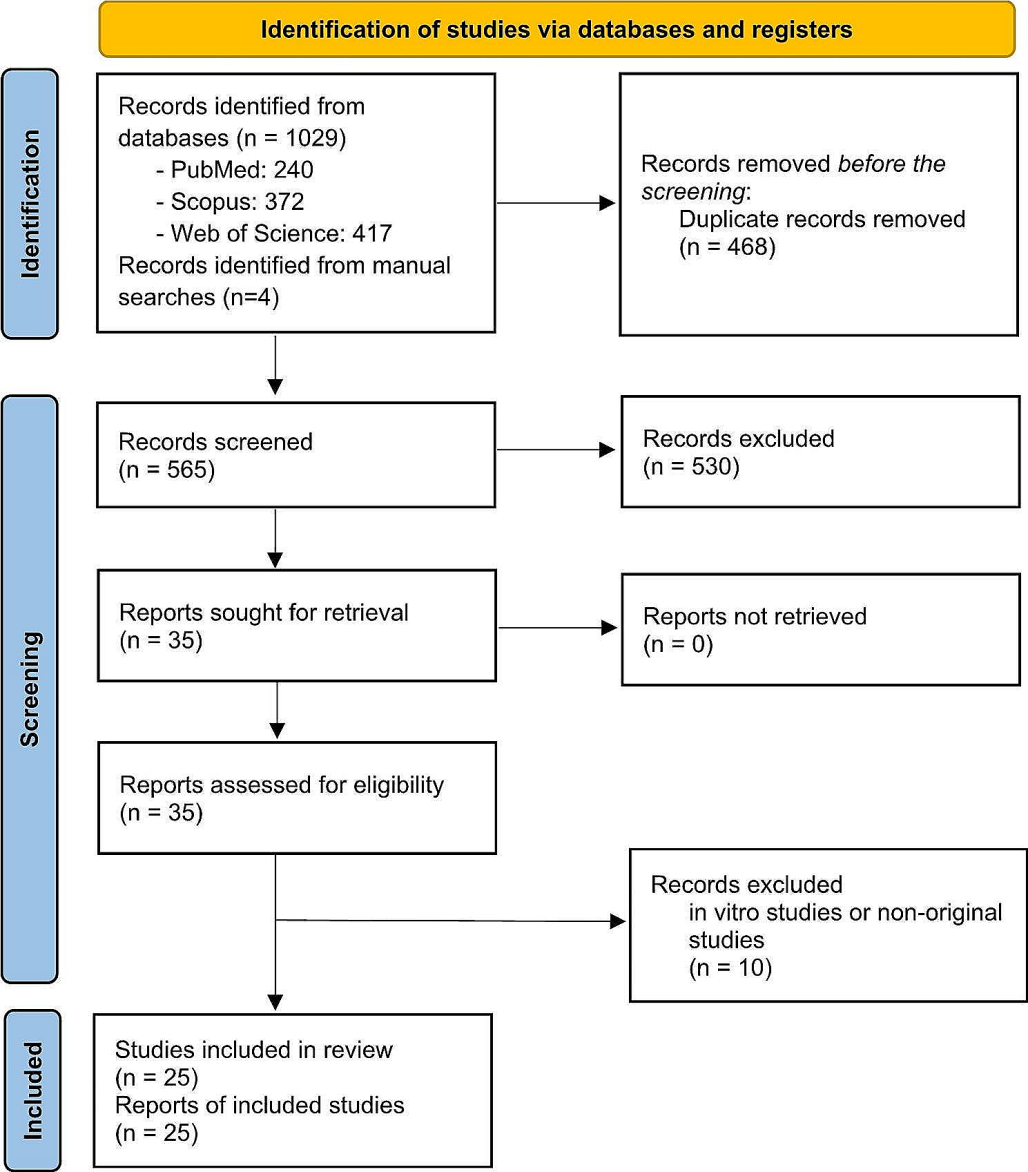
PRISMA 2020 flow diagram for new systematic reviews which included searches of databases and registers only From: Page MJ, McKenzie JE, Bossuyt PM, Boutron I, Hoffmann TC, Mulrow CD, et al. The PRISMA 2020 statement: an updated guideline for reporting systematic reviews. BMJ 2021;372:n71. doi: 10.1136/bmj.n71. For more information, visit: http://www.prisma-statement.org/

**Fig. 2:**
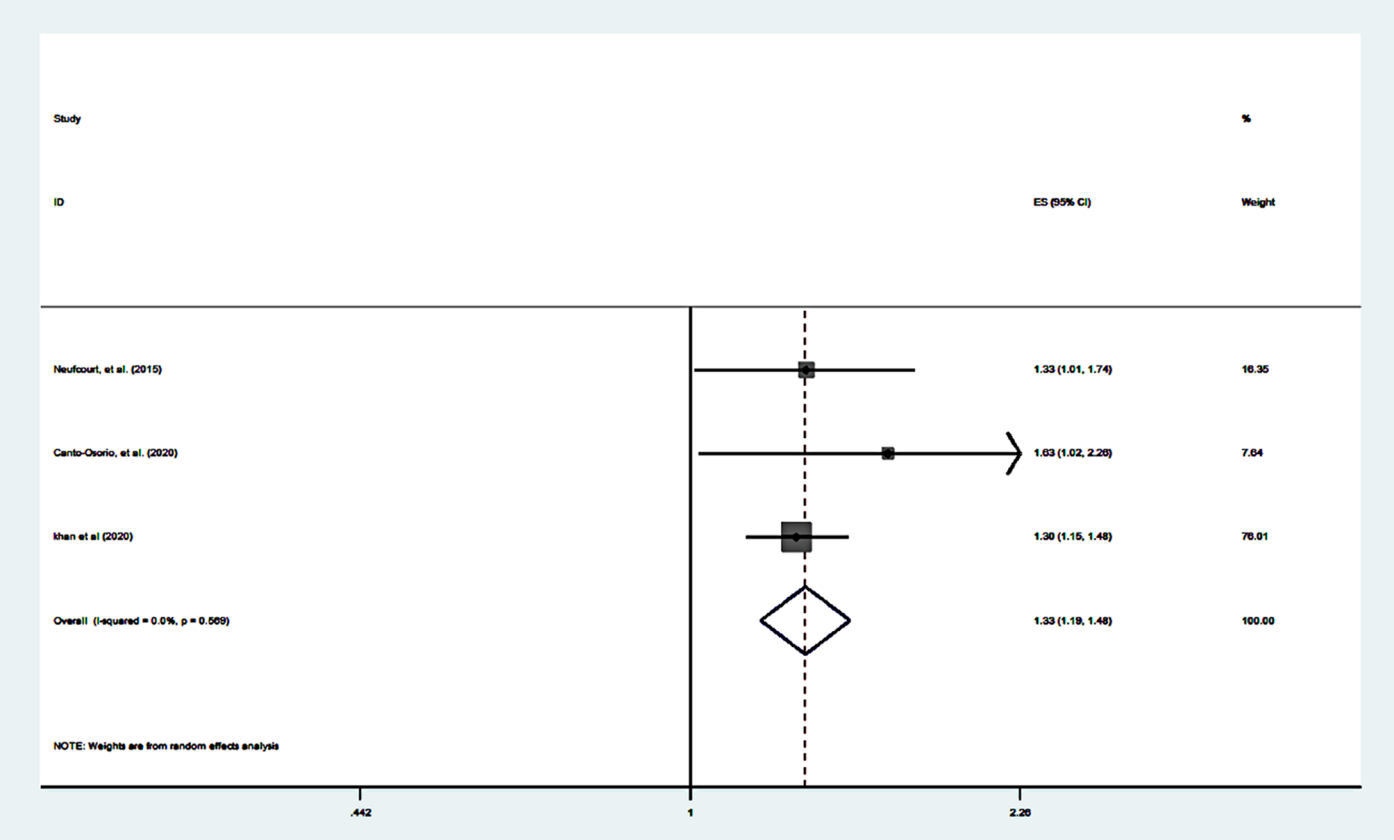
Forest plot of the association between the dietary inflammatory index and the risk of the metabolic syndrome in cohort studies. (top v. bottom category)

In cohort studies, the pooled OR for the association between the highest vs. lowest category of DII and MetS was OR = 1.33; 95% CI = 1.19–1.48) based on 3 data points (Table 1; Figs. 1, 2). We could not perform meta-analyses for MetS components in cohort groups due to a lack of information.

**Table 1:** Summary effects and 95% CI using random-effects meta-analysis for the associations of DII (top vs. bottom quartiles) with metabolic syndrome and its components, stratified by study characteristic^1^

groups and subgroups	number of data points	RR or OR (95% CI)	I^2^	*P*-value	
				Q test	subgroup difference
**Cohort studies**					
**Global analysis**					
Metabolic syndrome	3	1.33 (1.19–1.48)	0.0%	0.569	
**Cross-sectional studies**					
**Global analysis**					
Metabolic syndrome	24	1.24 (1.11–1.38)	73.9%	0.000	
Abdominal obesity	19	1.20 (0.99–1.46)	84.4%	0.000	
High blood pressure	18	1.19 (1.10–1.28)	27.0%	0.140	
Fasting blood sugar	18	1.18 (1.06–1.32)	57.0%	0.002	
HDL-C	17	1.09 (0.95–1.25)	68.1%	0.000	
Hypertriacylglyceamia	17	1.10 (0.97–1.25)	64.8%	0.000	
**Subgroup analysis**					
**Metabolic syndrome**					
**WHO region**					0.000
WPR	6	1.33 (1.20–1.47)	2.4%	0.401	
EUR	5	1.13 (0.99–1.30)	83.9%	0.000	
AMR	5	1.06 (1.0 -1.14)	80.1%	0.000	
EMR	6	1.36 (1.21–1.55)	36.3%	0.165	
SEAR	2	1.11 (1.02–1.22)	87.7%	0.004	
**MetS definition**					0.000
NCEP-ATP III	15	1.28 (1.20–1.37)	48.2%	0.019	
JIS	5	1.00 (0.93–1.08)	76.1%	0.002	
IDF	3	1.67 (1.29–2.16)	78.0%	0.011	
others	1	0.96 (0.77–1.19)	-	-	
**Adjusted for BMI**					0.001
Yes	16	1.25 (1.17–1.33)	76.5%	0.000	
No	8	1.06 (1.00-1.14)	46.0%	0.073	
**Adjusted for physical activity**					0.010
Yes	14	1.26 (1.17–1.37)	72.4%	0.000	
No	10	1.11 (1.05–1.18)	73.8%	0.000	
**Adjusted for energy intake**					0.083
Yes	7	1.24 (1.13–1.35)	77.6%	0.000	
No	17	1.13 (1.07–1.19)	72.6%	0.000	
**Adjusted for BMI, physical activity, and energy intake**					0.184
Yes	6	1.25 (1.11–1.40)	81.3%	0.000	
No	18	1.14 (1.09–1.20)	71.5%	0.000	
**Abdominal obesity**					
**Global analysis**	19	1.20 (0.99–1.46)	84.4%	0.000	
**Subgroup analysis**					
**WHO region**					0.000
WPR	5	1.27 (1.09–1.50)	66.7%	0.017	
EUR	4	1.04 (0.88–1.22)	59.2%	0.062	
AMR	2	1.26 (1.11–1.43)	8.8%	0.295	
EMR	6	1.99 (1.75–2.26)	89.5%	0.000	
SEAR	2	1.22 (0.93–1.59)	0.0%	0.400	
**MetS definition**					0.000
NCEP-ATP III	13	1.53 (1.41–1.65)	84.9%	0.000	
JIS	2	1.00 (0.75–1.32)	29.6%	0.233	
IDF	3	1.14 (0.91–1.44)	32.6%	0.227	
others	1	0.79 (0.61–1.03)	-	-	
**Adjusted for BMI**					0.050
Yes	13	1.43 (1.33–1.55)	88.5%	0.000	
No	6	1.21 (1.03–1.41)	32.8%	0.190	
**Adjusted for physical activity**					0.070
Yes	12	1.27 (1.13–1.43)	18.3%	0.264	
No	7	1.45 (1.33–1.58)	93.9%	0.000	
**Adjusted for energy intake**					0.037
Yes	7	1.28 (1.16–1.42)	0.0%	0.484	
No	12	1.49 (1.35–1.63)	89.6%	0.000	
**Adjusted for BMI, physical activity, and energy intake**					0.222
Yes	7	1.27 (1.09–1.48)	13.8%	0.324	
No	12	1.42 (1.31–1.53)	89.7%	0.000	
**Hypertension**					
**Global analysis**	18	1.19 (1.10–1.28)	27.0%	0.140	
**Subgroup analysis**					
**WHO region**					0.187
WPR	5	1.29 (1.15–1.44)	0.0%	0.898	
EUR	4	1.06 (0.94–1.21)	46.7%	0.131	
AMR	2	1.20 (1.02–1.42)	0.0%	0.845	
EMR	5	1.26 (1.11–1.44)	61.3%	0.035	
SEAR	2	1.12 (0.94–1.33)	0.0%	0.838	
**MetS definition**					0.566
NCEP-ATP III	12	1.22 (1.14–1.31)	0.0%	0.605	
JIS	2	1.16 (0.92–1.47)	70.6%	0.065	
IDF	3	1.19 (0.98–1.44)	76.9%	0.013	
others	1	1.05 (0.86–1.28)	-	-	
**Adjusted for BMI**					0.515
Yes	12	1.21 (1.13–1.30)	0.0%	0.509	
No	6	1.16 (1.03–1.30)	60.4%	0.027	
**Adjusted for physical activity**					0.585
Yes	11	1.17 (1.07–1.29)	48.7%	0.034	
No	7	1.22 (1.12–1.32)	0.0%	0.746	
**Adjusted for energy intake**					0.586
Yes	6	1.22 (1.11–1.35)	0.0%	0.651	
No	12	1.18 (1.09–1.28)	44.1%	0.050	
**Adjusted for BMI, physical activity, and energy intake**					0.610
Yes	5	1.23 (1.09–1.40)	0.0%	0.511	
No	13	1.19 (1.13–1.27)	39.2%	0.072	
**Hyperglycemia**					
**Global analysis**	18	1.18 (1.06–1.32)	57.0%	0.002	
**Subgroup analysis**					
**WHO region**					0.020
WPR	5	1.24 (1.11–1.39)	56.3%	0.057	
EUR	4	0.99 (0.87–1.13)	56.6%	0.074	
AMR	1	2.03 (1.08–3.82)	-		
EMR	6	1.24 (1.09–1.41)	38.5%	0.149	
SEAR	2	1.09 (0.93–1.28)	72.6%	0.056	
**MetS definition**					0.002
NCEP-ATP III	12	1.24 (1.15–1.33)	37.8%	0.089	
JIS	2	0.82 (0.66-1.00)	0.0%	0.684	
IDF	3	1.05 (0.86–1.29)	70.2%	0.035	
others	1	1.11 (0.91–1.35)	-	-	
**Adjusted for BMI**					0.007
Yes	12	1.10 (1.02–1.18)	61.6%	0.003	
No	6	1.34 (1.18–1.52)	0.0%	0.613	
**Adjusted for physical activity**					0.737
Yes	12	1.14 (1.05–1.25)	60.7%	0.003	
No	6	1.17 (1.06–1.29)	56.1%	0.044	
**Adjusted for energy intake**					0.558
Yes	6	1.13 (1.01–1.26)	66.0%	0.012	
No	12	1.17 (1.08–1.27)	55.1%	0.011	
**Adjusted for BMI, physical activity, and energy intake**					0.737
Yes	7	1.14 (1.02–1.27)	63.9%	0.011	
No	11	1.17 (1.08–1.26)	56.1%	0.012	
**Low HDL-C**					
**Global analysis**	17	1.09 (0.95–1.25)	68.1%	0.000	
**Subgroup analysis**					
**WHO region**					0.000
WPR	4	1.16 (0.99–1.37)	0.0%	0.563	
EUR	4	0.89 (0.75–1.06)	81.9%	0.001	
AMR	1	1.03 (0.58–1.81)	-	-	
EMR	6	1.27 (1.16–1.40)	36.4%	0.164	
SEAR	2	0.88 (0.75–1.02)	0.0%	0.584	
**MetS definition**					0.000
NCEP-ATP III	11	1.17 (1.08–1.26)	62.8%	0.003	
JIS	2	1.11 (0.89–1.39)	0.0%	0.561	
IDF	3	1.08 (0.80–1.45)	0.0%	0.536	
others	1	0.62 (0.48–0.80)	-	-	
**Adjusted for BMI**					0.385
Yes	10	1.09 (1.02–1.18)	79.9%	0.00	
No	7	1.18 (1.01–1.38)	0.0%	0.592	
**Adjusted for physical activity**					0179
Yes	11	1.05 (0.96–1.16)	49.7%	0.030	
No	6	1.16 (1.06–1.26)	82.4%	0.000	
**Adjusted for energy intake**					0.177
Yes	6	1.04 (0.92–1.17)	68.1%	0.008	
No	11	1.14 (1.06–1.24)	69.4%	0.000	
**Adjusted for BMI, physical activity, and energy intake**					0.163
Yes	5	1.03 (0.92–1.16)	74.3%	0.004	
No	12	1.14 (1.04–1.18)	66.3%	0.001	
**Hypertriacylglcerolemia**					
**Global analysis**	17	1.10 (0.97–1.25)	64.8%	0.000	
**Subgroup analysis**					
**WHO region**					0.001
WPR	4	1.25 (1.10–1.43)	9.7%	0.344	
EUR	4	1.12 (0.97–1.29)	0.0%	0.521	
AMR	1	0.77 (0.42–1.42)	-	-	
EMR	6	0.89 (0.80–0.98)	74.4%	0.002	
SEAR	2	1.15 (0.97–1.36)	0.0%	0.445	
**MetS definition**					0.380
NCEP-ATP III	11	1.03 (0.95–1.11)	75.0%	0.000	
JIS	2	1.02 (0.82–1.27)	0.0%	0.929	
IDF	3	1.25 (1.02–1.52)	15.5%	0.306	
others	1	1.04 (0.84–1.29)	-	-	
**Adjusted for BMI**					0.002
Yes	10	0.99 (0.92–1.07)	69.3%	0.001	
No	7	1.25 (1.10–1.42)	11.5%	0.342	
**Adjusted for physical activity**					0.000
Yes	11	1.21 (1.11–1.33)	2.0%	0.422	
No	6	0.90 (0.82–0.99)	68.1%	0.008	
**Adjusted for energy intake**					0.007
Yes	6	1.21 (1.07–1.36)	4.6%	0.387	
No	11	0.99 (0.92–1.07)	69.6%	0.000	
**Adjusted for BMI, physical activity, and energy intake**					0.14
Yes	5	1.19 (1.06–1.35)	9.8%	0.352	
No	12	1.00 (0.92–1.08)	68.5%	0.000	

^1^DII: Dietary Inflammatory Index; EMR, Eastern Mediterranean Region; AMR, Americas Region; EUR, European Region; SEAR, South-East Asia Region; WPR, Western Pacific Region; NCEP-ATPIII, The Third Report of the National Cholesterol Education Program Expert Panel on Detection, Evaluation, and Treatment of High Blood Cholesterol in Adults (Adult Treatment Panel III); IDF, The International Diabetes Federation Criteria; JIS, Joint Interim Statement; MetS: Metabolic syndrome

**Fig. 3:**
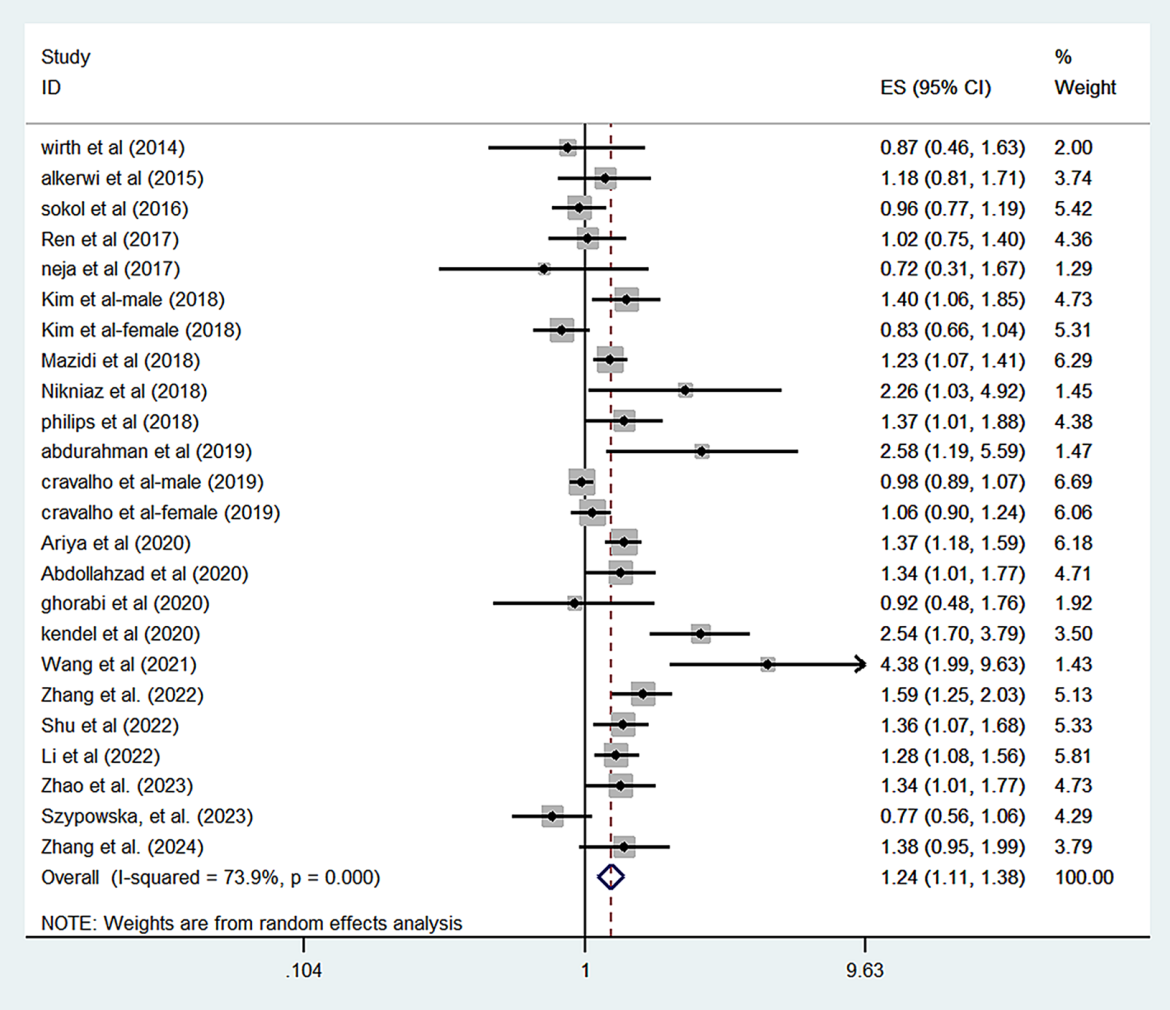
Forest plot of the association between the dietary inflammatory index and the risk of the metabolic syndrome in cross-sectional studies. (top v. bottom category)

In cross-sectional studies, the pooled OR for the association between the highest vs. lowest category of DII and MetS was 1.24 (95% CI = 1.11–1.38), with significant heterogeneity between studies (I^2^ = 73.9%, *P* < 0.001) based on 24 data points (Table 1; Fig. 3). Individuals in the highest category of DII had significantly higher odds of hypertension (OR = 1.19; 95% CI = 1.10–1.28; I^2^ = 27.0%, *P* = 0.14) and hyperglycemia (OR = 1.19; CI = 1.06–1.32; I^2^ = 57.0%, *P* = 0.002). However, the pooled OR comparing the top vs. bottom category of DII with risk of abdominal obesity (OR = 1.20; 95% CI = 0.99–1.46; I^2^ = 84.4%, *P* < 0.001), low HDL-C (OR = 1.09; 95% CI = 0.95–1.25; I^2^ = 68.1%, *P* < 0.001), and hypertriglyceridemia (OR = 1.10; 95% CI = 0.97–1.25; I^2^ = 64.8%, *P* < 0.001) were not statistically significant (Table 1 and Supplemental Figs. 1–5).

No evidence of publication bias was detected in the meta-analyses for MetS and its components according to funnel plot and Egger’s tests in both cohort and cross-sectional studies (Supplemental Figs. 6–12).

In total, 11 cross-sectional studies with 41,783 participants and 12,310 MetS cases were included in the dose-response analysis. In the dose-response meta-analysis, there was no significant non-linear association between DII and MetS (P for non-linearity = 0.524). The pooled OR for a 1-unit increase in DII was significantly associated with the MetS in a linear dose-response analysis (OR = 1.044, 95% CI = 1.01–1.07, *P* = 0.003). For the MetS components, 7 cross-sectional studies with a total of 20,058 participants were included in the dose-response analysis. There was no significant non-linear or linear association between DII and MetS components except for FBS (OR = 1.11, 95% CI = 1.05–1.17, P for non-linearity < 0.001). More details can be found in Supplemental Figs. 13–18. We were not able to perform a dose-response meta-analysis for the cohort studies due to a lack of information.

### Subgroup analyses and sensitivity analyses

Table 1 shows the detailed results of the subgroup analysis of the components of the metabolic syndrome. Due to the lack of sufficient information, we were not able to perform a subgroup analysis for the cohort studies. In a subgroup of the meta-analysis of cross-sectional studies, significant associations between the DII (upper and lower quartiles) and the MetS were found in different WHO regions, except for the European Region. Moreover, DII (top vs. bottom quartiles) and MetS were significantly associated when MetS was defined according to NCEP-ATP III and IDF criteria. Also, after adjustment for BMI, physical activity, total energy intake, or any combination of these covariates, DII (top vs. bottom quartiles) was significantly associated with MetS, abdominal obesity, hypertension, hyperglycemia, and hypertriglyceridemia.

Sensitivity analyses showed that the overall effect size of the association between DII and the risk of MetS did not depend on the individual study for cohort studies (CI range: 1.13–1.78) and cross-sectional studies (CI range: 1.09–1.41) (Supplemental Figs. 19 and 20).

## Discussion

The present meta-analysis used data from all available observational studies, including cohort and cross-sectional studies, to investigate the association between inflammatory characteristics of diet and MetS and its components. According to the current meta-analysis of cohort and cross-sectional studies, higher DII was positively associated with the risk of MetS. In addition, according to 22 cross-sectional studies, a pro-inflammatory dietary pattern is associated with hyperglycemia and hypertension. However, subgroup analysis revealed that after adjustment for BMI, physical activity, total energy intake, or any combination of these covariates, a higher DII score was significantly associated with MetS, abdominal obesity, hypertension, hyperglycemia, and hypertriglyceridemia.

Given that the DII is derived from up to 45 dietary components and potentially represents the inflammatory status of a diet [10], it is expected that a high DII score, which stimulates or modulates chronic and systemic inflammation, is associated with a higher risk of chronic disease [12]. Although studies in this area are inconsistent. To date, several reviews and meta-analyses have examined the association between DII, as indicated more pro-inflammatory diet, and cardiometabolic diseases such as MetS, CVD, cancer, and mortality [15, 45–47]. A recent meta-analysis of 60 prospective cohorts and 67 case-control studies suggested that dietary inflammatory potential may be associated with a higher risk of CVD, colorectal cancer, and all-cause mortality [45]. Also, several studies showed that a higher DII score is generally associated with the risk of CVD, CVD-related mortality, and obesity [15, 46, 47], whereas the relationship between DII and MetS is less consistent [15, 16, 47]. Our study reflects the findings of a meta-analysis of 18 observational studies published recently that assessed the association between DII and MetS and its components [16]. However, other meta-analyses have not found an association between DII and the risk of MetS [15, 47]. These conflicting results may be due to the number of studies included in the previous meta-analysis. For example, only five studies (two cohort studies and three cross-sectional studies) were included in the meta-analysis by Namazi et al. [15]. However, it is remarkable to note that even with a null association, Namazi et al. [15] found a significant association between the DII and some components of MetS. The link between the inflammatory potential of the diet and its effect on the characteristics of the metabolic syndrome needs to be further confirmed, in particular in longitudinal studies.

According to our subgroup analysis, despite the dietary variables measured by the different instruments used to calculate the DII, increased dietary inflammatory potential remained significantly associated with MetS risk. Nonetheless, significant associations were only observed when MetS was defined according to NCEPATP III and IDF criteria. As a result of these findings, future meta-analyses on MetS should rely on a consistent and widely accepted definition. In addition, our subgroup results showed that the association between DII and MetS risk was significant in the different WHO regions, except in the European Region. It is possible to observe such differences between geographical regions and ethnicities because DII can be affected by different dietary patterns [48]. People in the American population and other regions that are undergoing a nutritional transition tend to consume Western dietary patterns with high-containing pro-inflammatory foods, whereas people in different European countries, such as the Mediterranean region, tend to consume healthy foods such as fruit, vegetables, and seafood more often [48]. There is still a need for evidence on the links between a pro-inflammatory diet and the development of the MetS and its components in different regions and population groups.

A growing body of research has demonstrated the associations between the inflammatory properties of foods and inflammatory biomarkers [47]. MetS is associated with elevated inflammatory biomarkers such as TNF-α, CRP, and interleukins (known as IL-1β, IL-4, IL-6, and IL-10) and were considered in the development of the DII [10]. Inflammatory factors can lead to lipid abnormalities and insulin resistance, which may lead to the development of MetS and CVD [49]. Furthermore, obesity and its consequences can lead to an imbalanced adipokine profile, increasing levels of proinflammatory adipokines and cytokines including retinol-binding protein 4, lipocalin 2, Leptin, and chemerin [50].

Some studies have revealed an inverse association between serum concentration of inflammatory biomarkers and healthy dietary patterns [51, 52]. The beneficial effects of the healthy dietary pattern on metabolic disorders may be due to the high consumption of fruits, vegetables, whole grains, legumes, nuts, low-fat dairy products, fish, and olive oil and the low consumption of alcohol, red and processed meat, simple carbohydrate, and saturated fatty acids [51]. A Mediterranean diet as a healthy dietary pattern can modulate systemic inflammation and attenuate the progression and development of inflammatory diseases through improved serum concentration of CRP, and IL-6, as well as endothelial function parameters such as intercellular adhesion molecule-1 [7]. Results from the Nurses’ Health Study and Health Professionals Follow-up Study indicated that following a prudent dietary pattern, including a high intake of whole grains, legumes, fruits, vegetables, poultry, and fish, was associated with an inverse association with insulin, homocysteine, CRP, and E-selectin [53].

Several positive effects of the Mediterranean diet can be attributed to phytochemicals and nutrients such as fiber, omega-3 polyunsaturated fatty acids, polyphenols, vitamin C, vitamin E, and magnesium [54]. The nutrients mentioned above have low pro-inflammatory properties, prevent inflammation, and act as antioxidants, inhibiting the production of free radicals. Conversely, unhealthy dietary patterns with a high intake of pro-inflammatory foods (red and processed meats, refined carbohydrates, and saturated fatty acids) can lead to an increase in inflammatory cytokines such as E-selectin, soluble vascular cell adhesion molecule-1, and hs-CRP [51, 52].

The current study presents the most updated and comprehensive assessment to date of the association between DII and MetS. We also explored the association between DII and individual MetS components separately because of the relatively greater quantity of data. In this meta-analysis, we included 25 unique articles which is a considerable improvement compared to the recent meta-analysis of 18 articles conducted by Yi et al. [16]. However, the current study has several limitations. One of the limitations of this review is the heterogeneity of the studies, such as sample size, study design, follow-up periods, and characteristics of the study population. Although there was considerable heterogeneity between the studies, subgroup analyses were performed to determine the source of heterogeneity. Twenty-two of the 25 included studies in this systematic review and meta-analysis had a cross-sectional design, which did not provide temporal associations. However, despite the limited number of cohort studies (three for MetS), the association of DII with MetS remained strong and positive, as did a large number of cross-sectional studies. Also, the food assessment questionnaires were different and were therefore a source of recall bias. Furthermore, as the diets of adults change over time, the baseline DII score used by the studies may not be representative of the true long-term dietary pattern.

## Conclusion

In summary, the results of this systematic review and meta-analysis of observational studies indicate that higher DII is positively associated with MetS in both cohort and cross-sectional designs. In the current meta-analysis of cross-sectional studies, higher DII was also significantly associated with abdominal obesity, hypertension, hyperglycemia, and hypertriglyceridemia after adjustment for BMI, physical activity, total energy intake, or any combination of these covariates. Based on the results of this study, a pro-inflammatory diet plays an important role in developing MetS, supporting the need for dietary interventions to prevent it.

The evidence on the relationship between the DII and MetS and its components is contradictory and limited. The limitations of the evidence are that it is mostly based on observational studies, such as cross-sectional studies, so causality cannot be established with certainty. So, further evidence is needed from future studies, including prospective studies, randomized controlled trials, and adaptive/pragmatic studies. Future studies could determine how a DII-compliant dietary intervention (i.e. aimed at lowering the overall DII) would help prevent new-onset MetS in high-risk populations and improve metabolic dysfunction in patients with MetS.

### Electronic supplementary material

Below is the link to the electronic supplementary material.

Supplementary Material 1

Supplementary Material 2

## Data Availability

No datasets were generated or analysed during the current study.
