# Supplementary material for: Dietary inflammatory index and its association with risk of metabolic syndrome and its components: a systematic review and Meta-analysis of Observational studies
Source: J Health Popul Nutr. 2024 Jun 19;43:87. doi: 10.1186/s41043-024-00580-w (PMC11188268; doi:10.1186/s41043-024-00580-w)
Supplement: Supplementary file 1 — Supplementary Material 1 [file 41043_2024_580_MOESM1_ESM.docx]

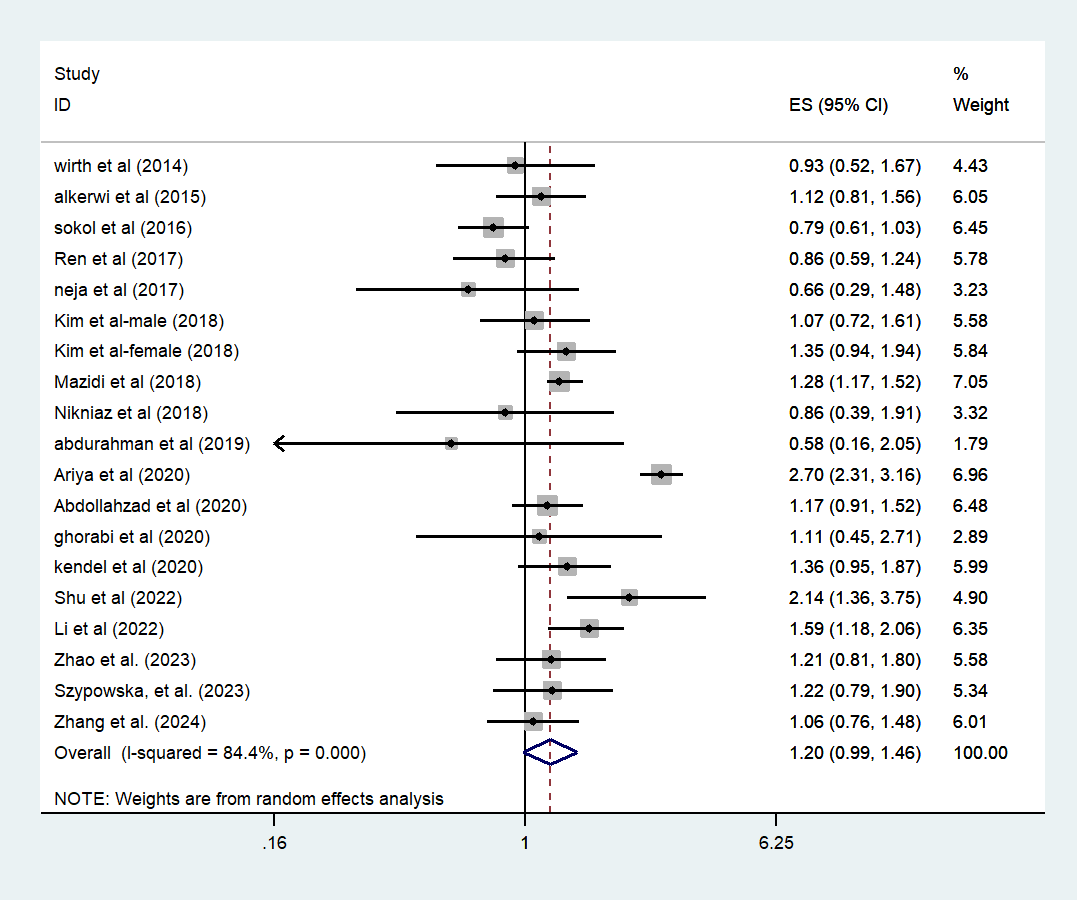


**Figure S1.** Forest plot of the association between the dietary inflammatory index and abdominal obesity in cross-sectional studies (top v. bottom category).


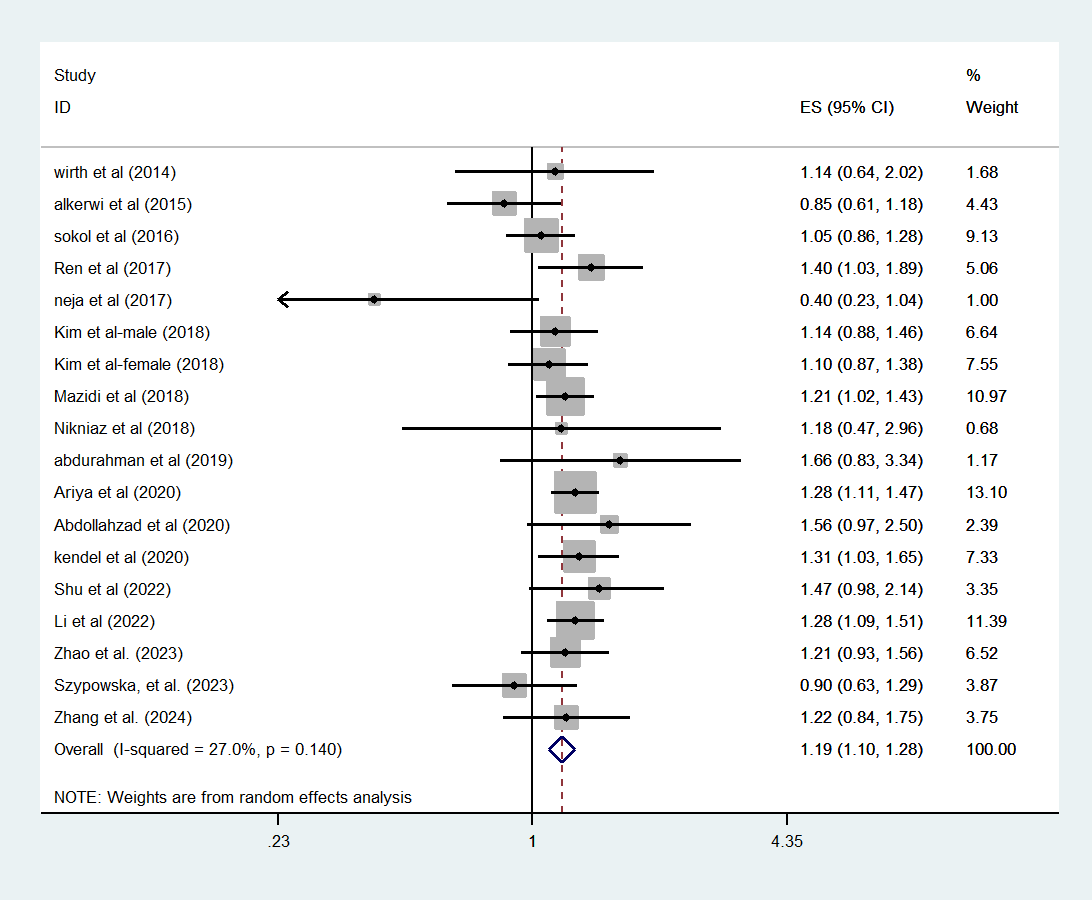


**Figure S2.** Forest plot of the association between the dietary inflammatory index and hypertension in cross-sectional studies (top v. bottom category).


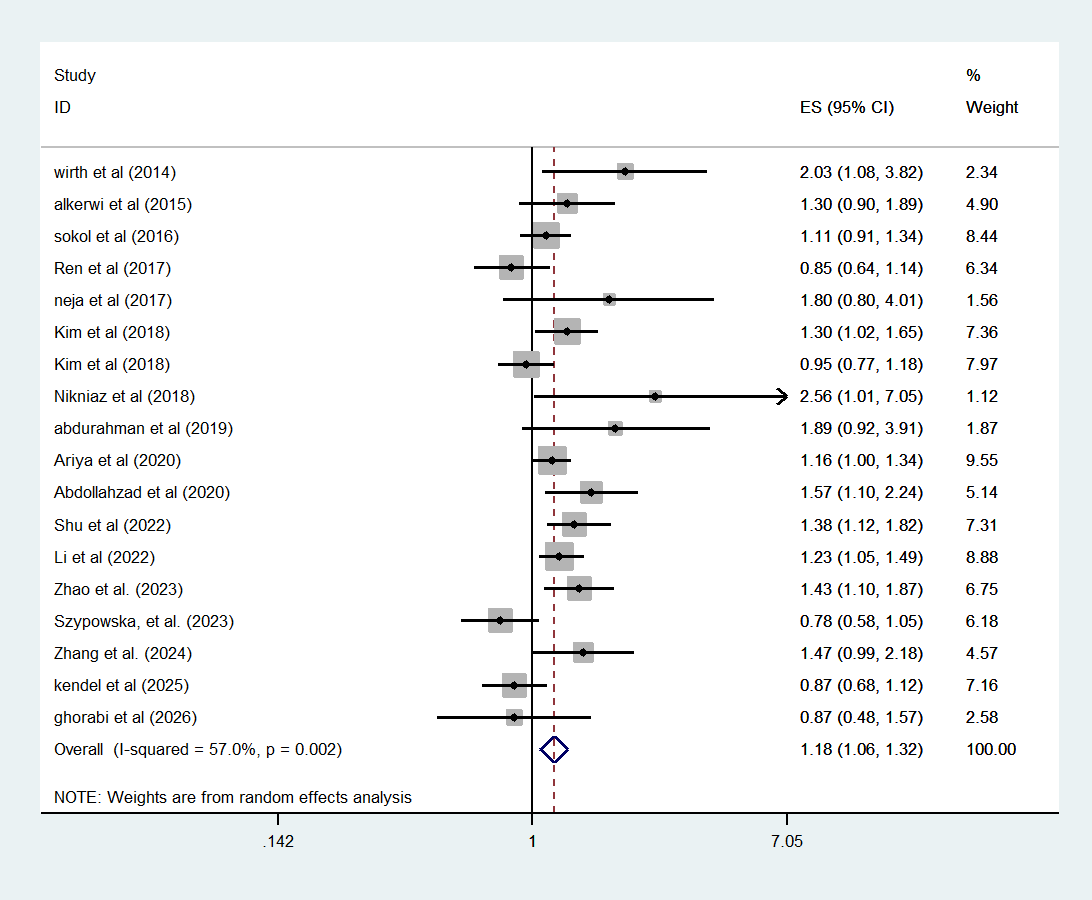


**Figure S3.** Forest plot of the association between the dietary inflammatory index and hyperglycemia in cross-sectional studies (top v. bottom category).


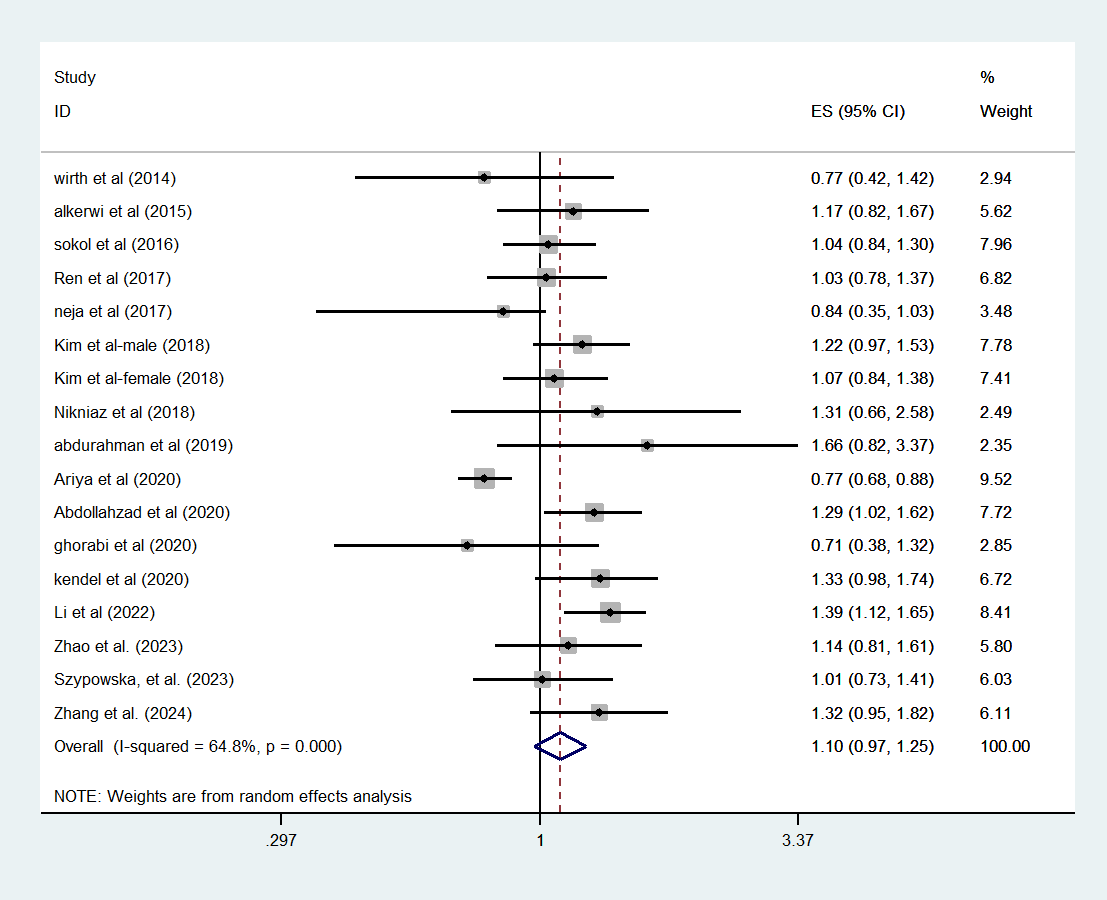


**Figure S4.** Forest plot of the association between the dietary inflammatory index and hypertriglyceridemia in cross-sectional studies (top v. bottom category).


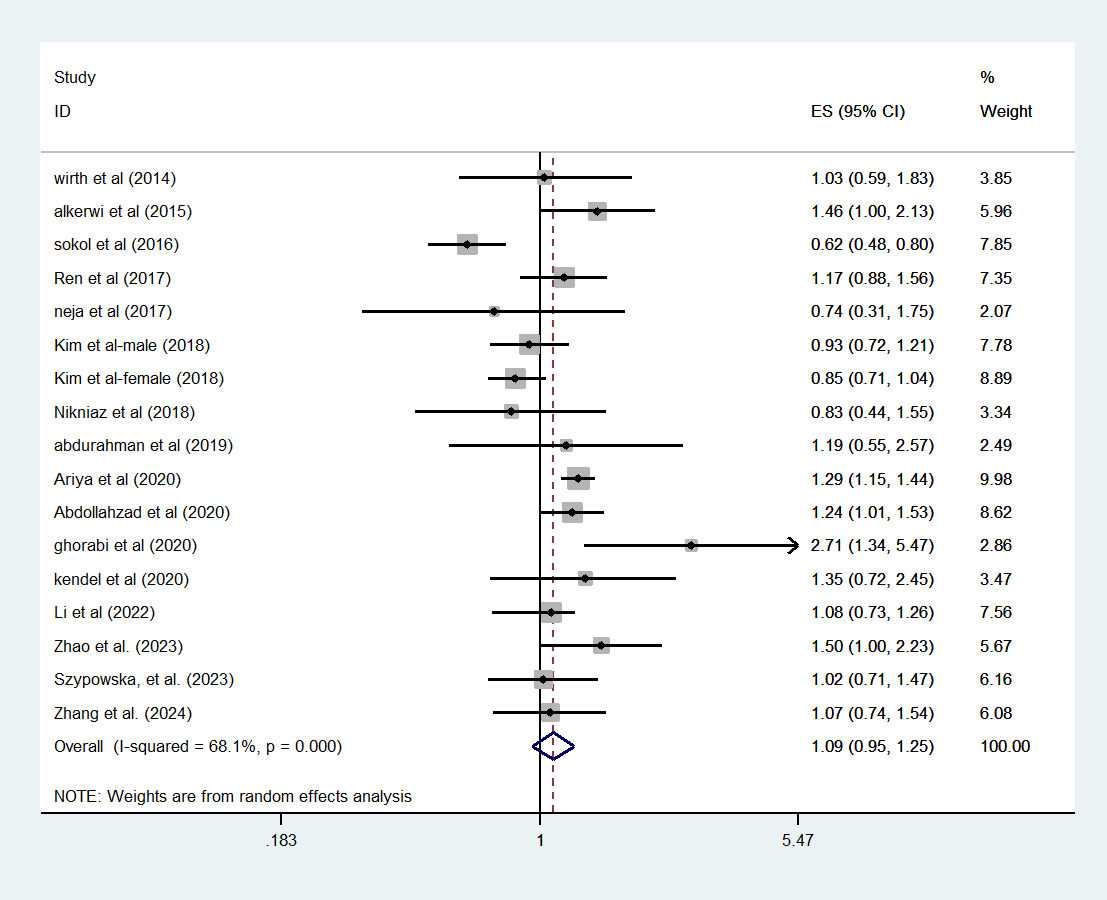


**Figure S5.** Forest plot of the association between the dietary inflammatory index and low HDL-cholesterol in cross-sectional studies (top v. bottom category).


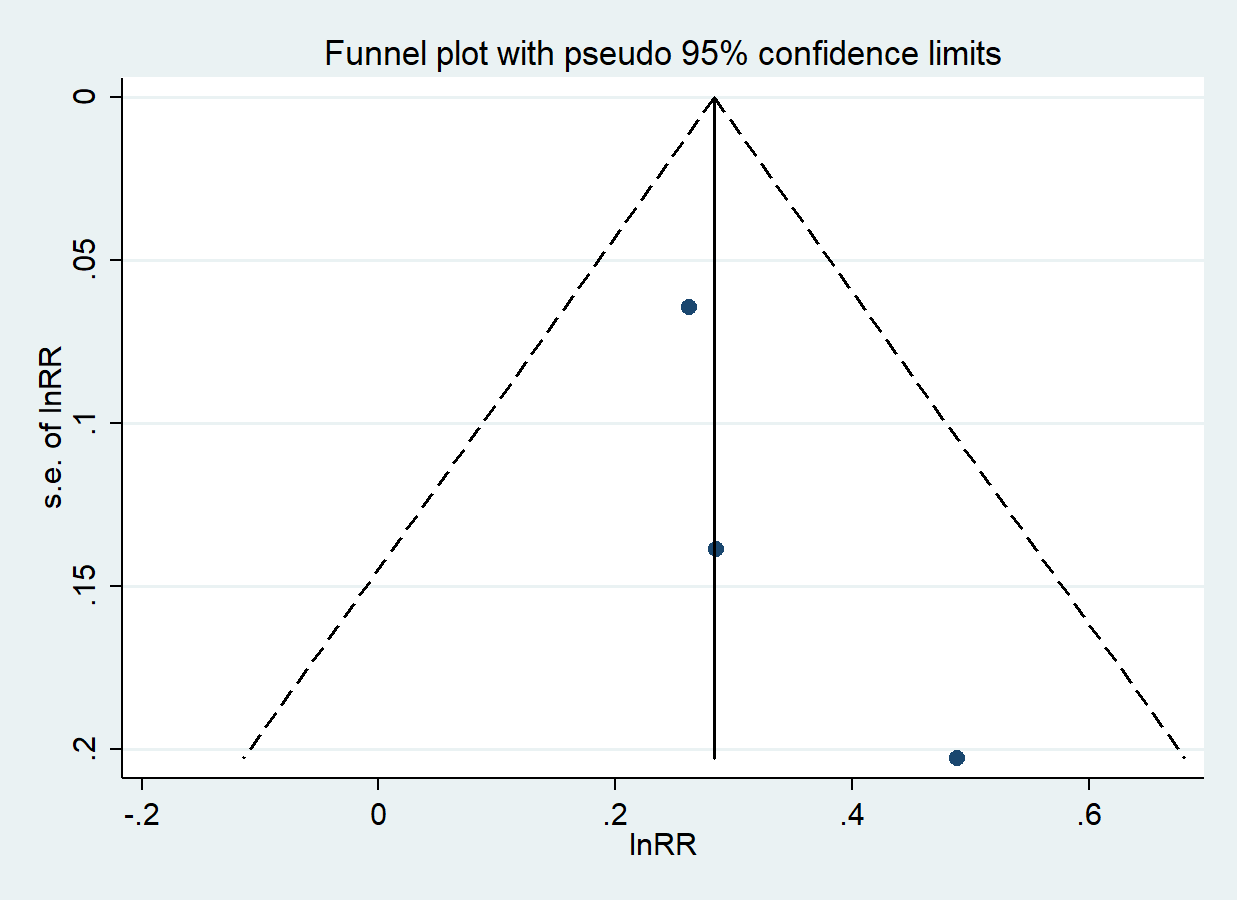


**Figure S6.** Funnel plot of publication bias for the association between DII (top vs. bottom quartiles) with MetS in cohort studies.

*Note: the results of Egger’s test were t=1.65, p=0.347*


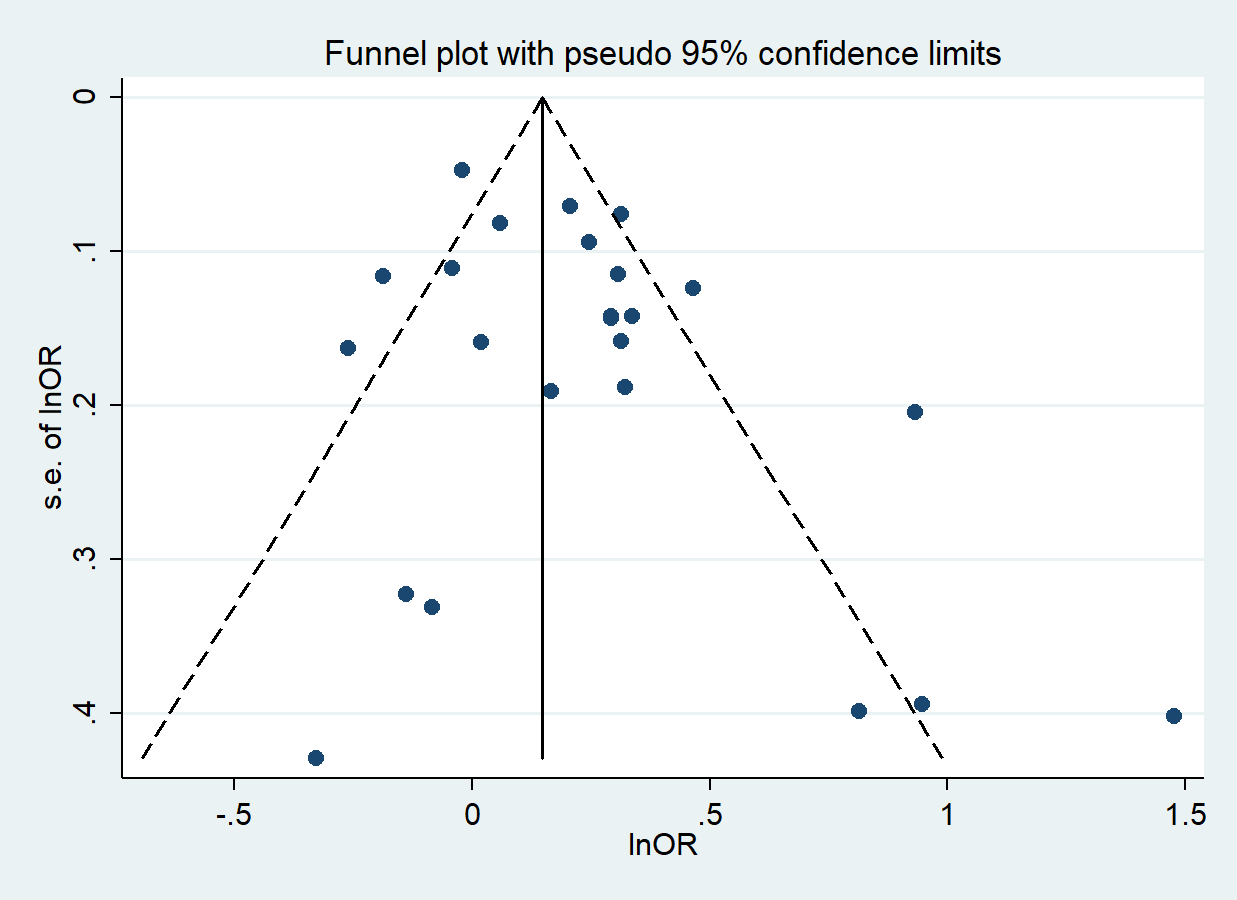


**Figure S7.** Funnel plot of publication bias for the association between DII (top vs. bottom quartiles) with MetS in cross-sectional studies.

*Note: the results of Egger’s test were t=1.97, p=0.062*


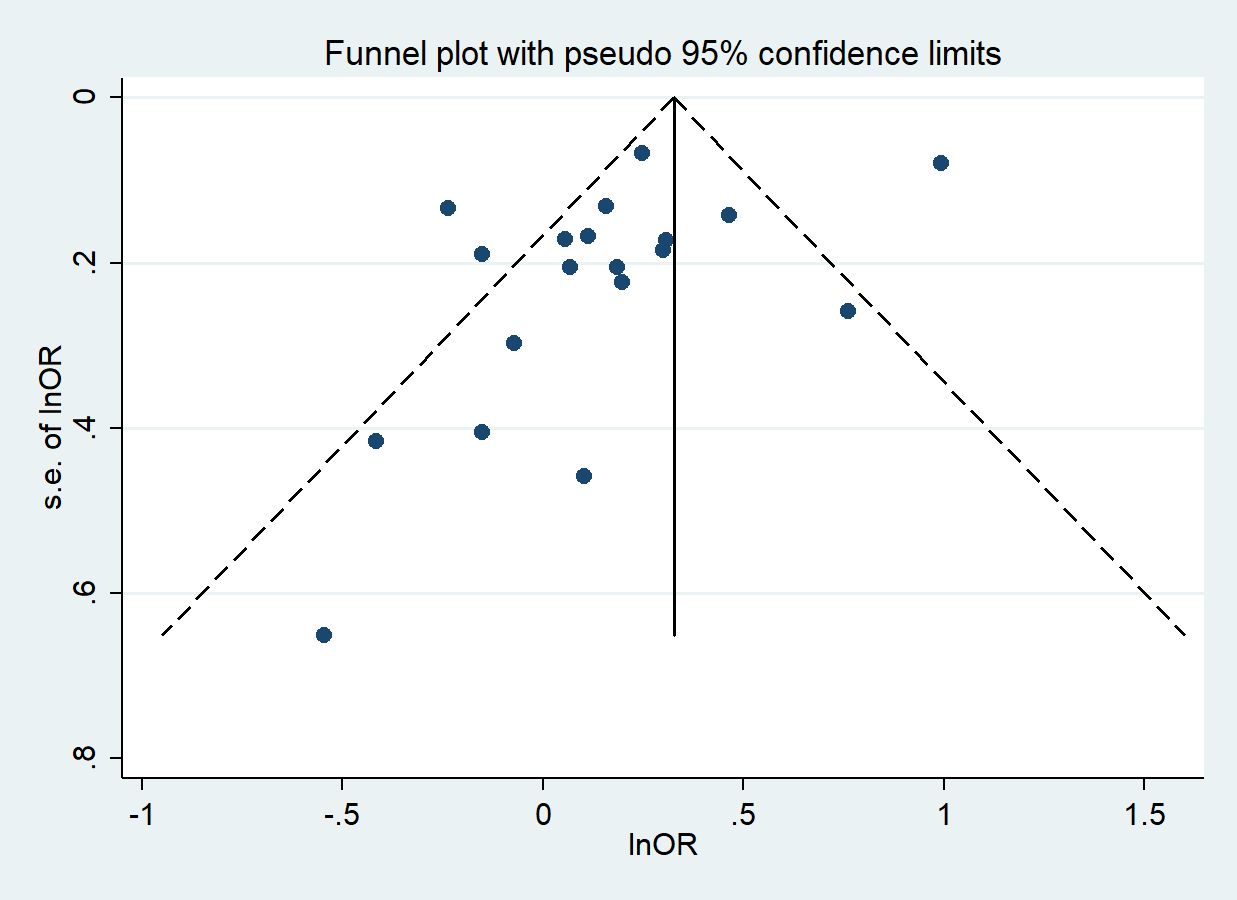


**Figure S8.** Funnel plot of publication bias for the association between DII (top vs. bottom quartiles) with abdominal obesity in cross-sectional studies.

*Note: the results of Egger’s test were t= -1.94, p=0.069*


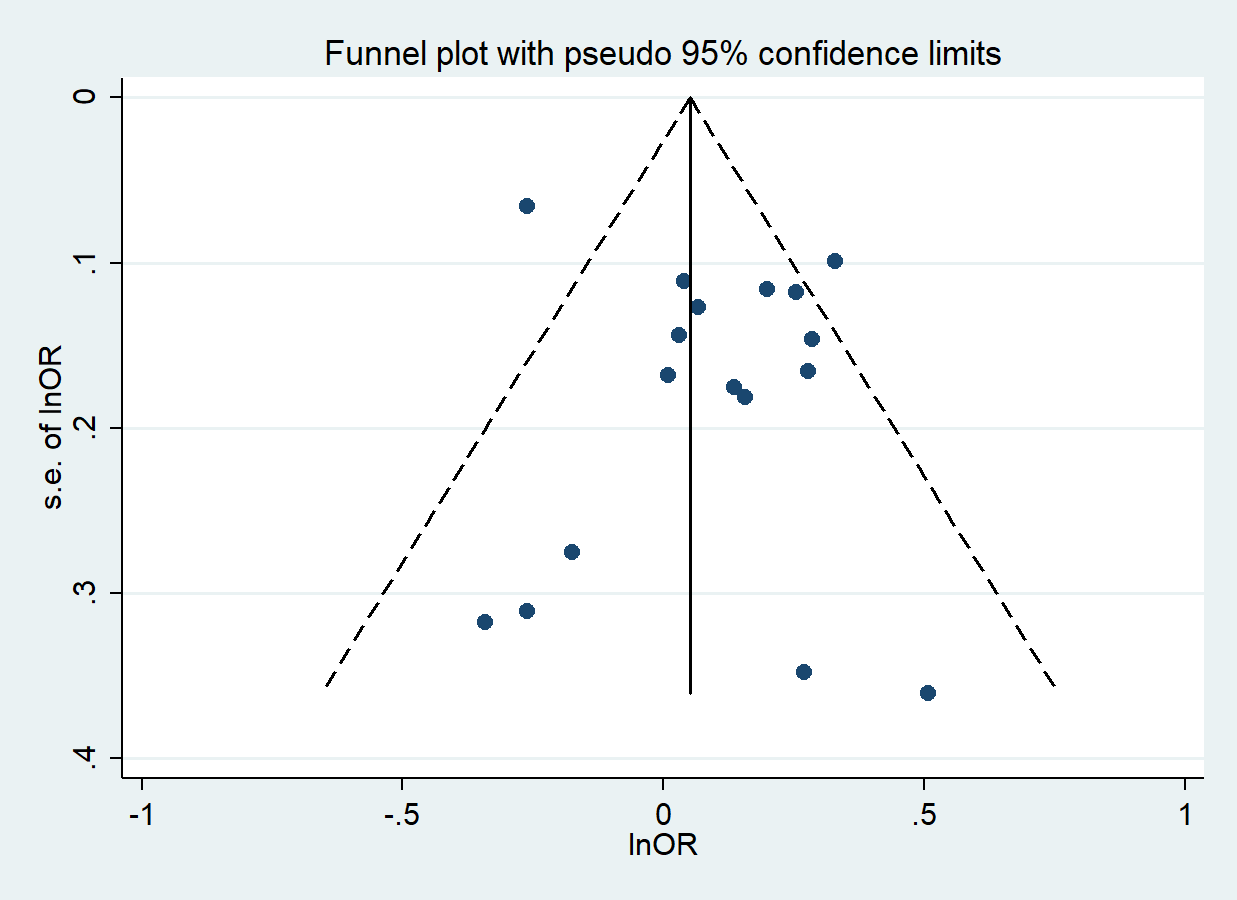


**Figure S9.** Funnel plot of publication bias for the association between DII (top vs. bottom quartiles) with hyperglycemia in cross-sectional studies.

*Note: the results of Egger’s test were t=1.23, p=0.239*


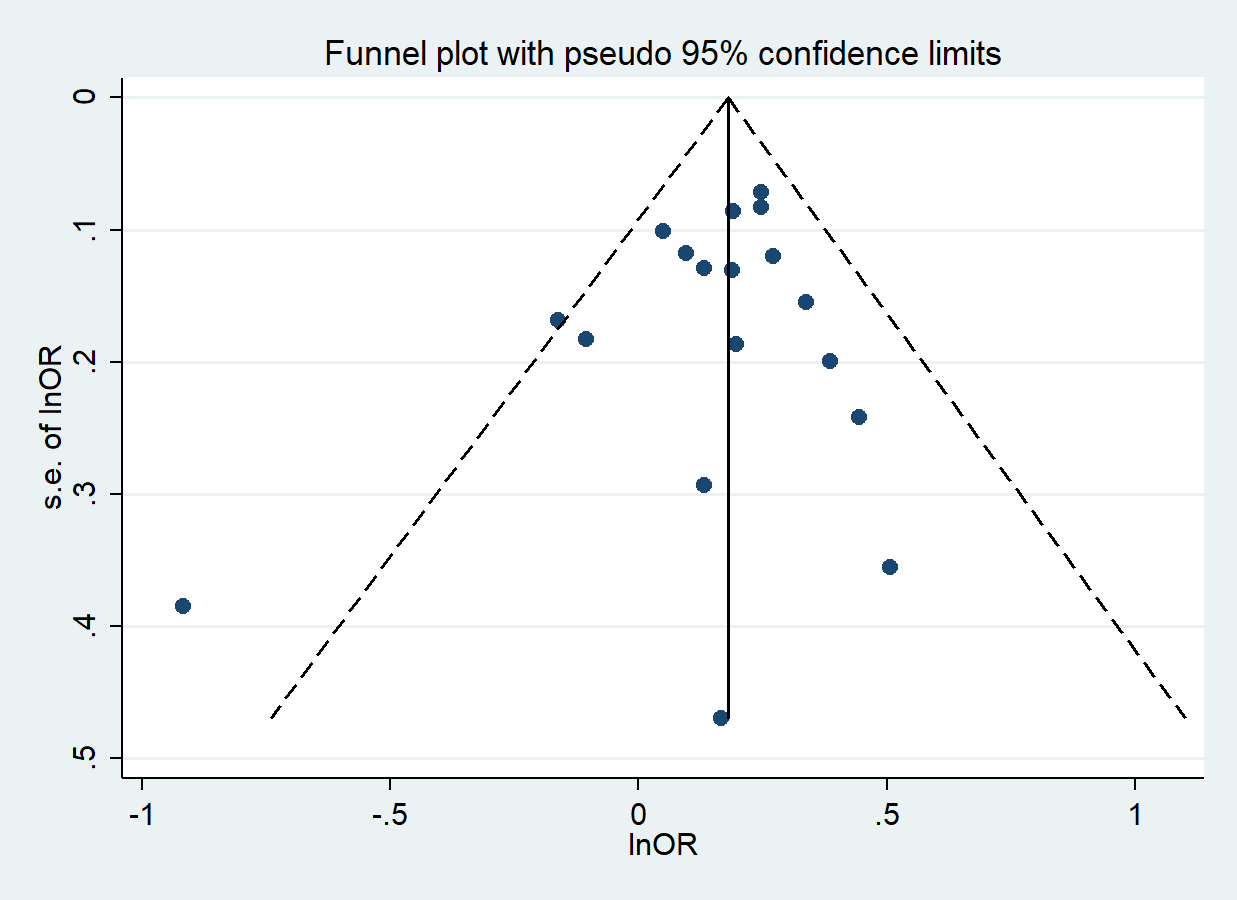


**Figure S10.** Funnel plot of publication bias for the association between DII (top vs. bottom quartiles) with hypertension in cross-sectional studies.

*Note: the results of Egger’s test were t= -1.00, p=0.331*


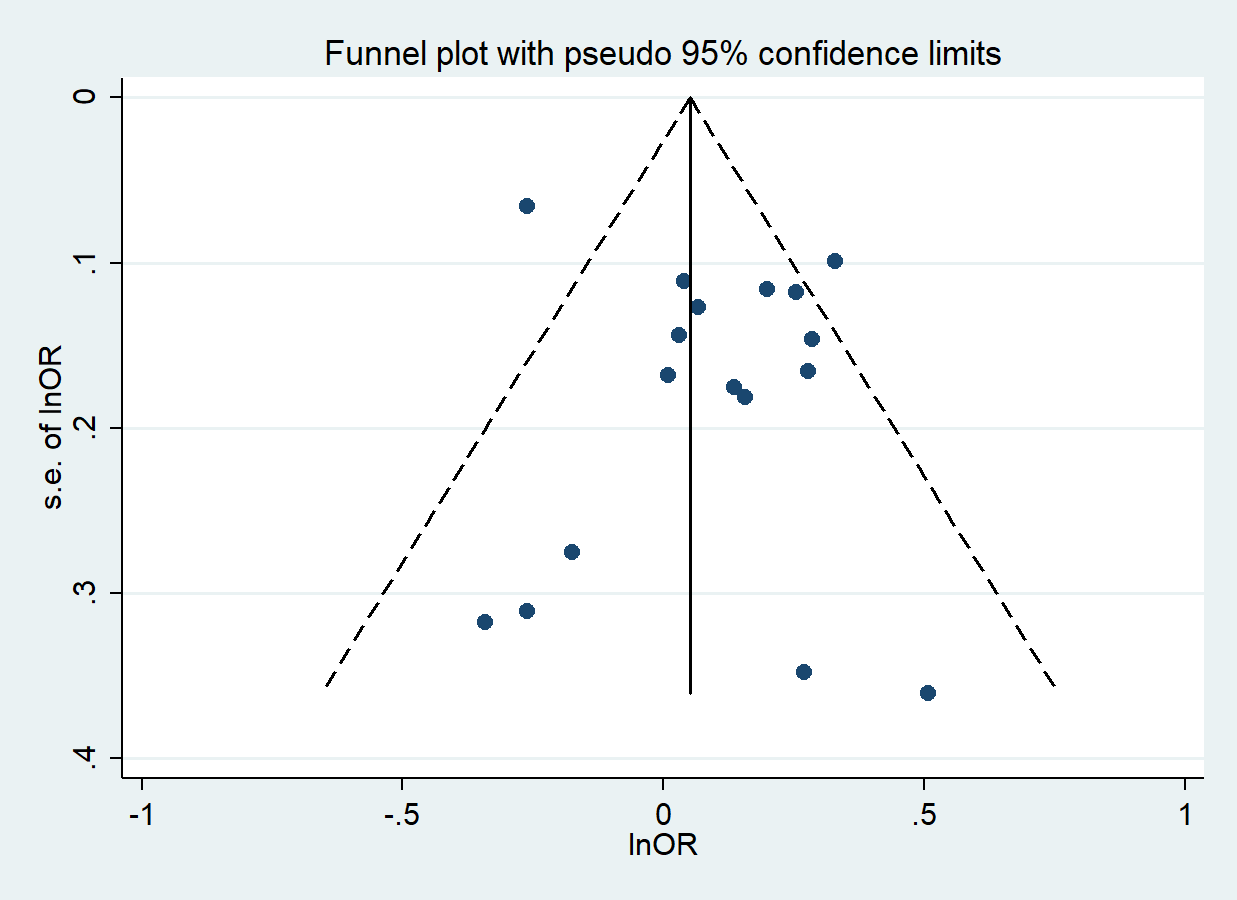


**Figure S11.** Funnel plot of publication bias for the association between DII (top vs. bottom quartiles) with hypertriglyceridemia in cross-sectional studies.

*Note: the results of Egger’s test were t= 1.23, p=0.239*


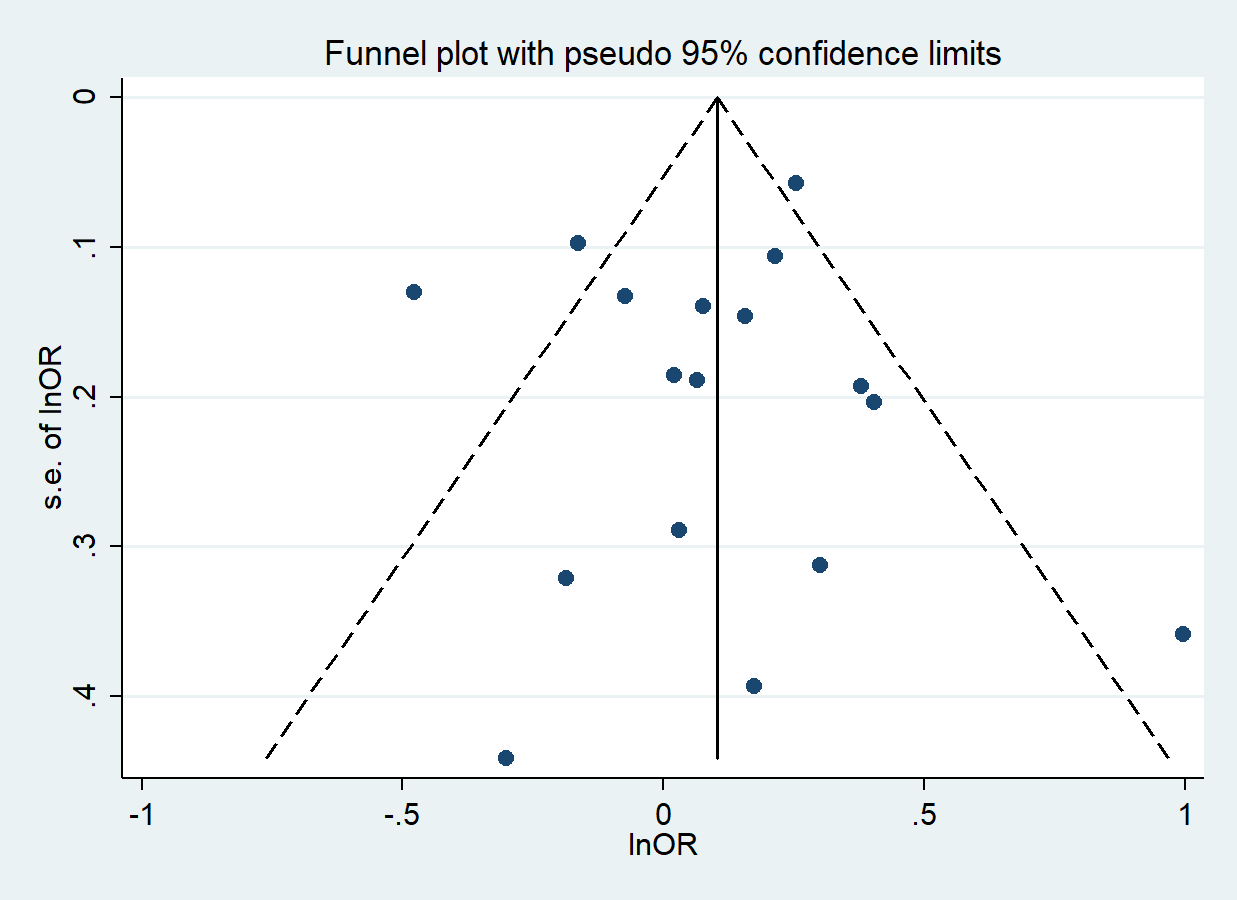


**Figure S12.** Funnel plot of publication bias for the association between DII (top vs. bottom quartiles) with low HDL-cholesterol

*Note: the results of Egger’s test were t=-0.29, p=0.775*


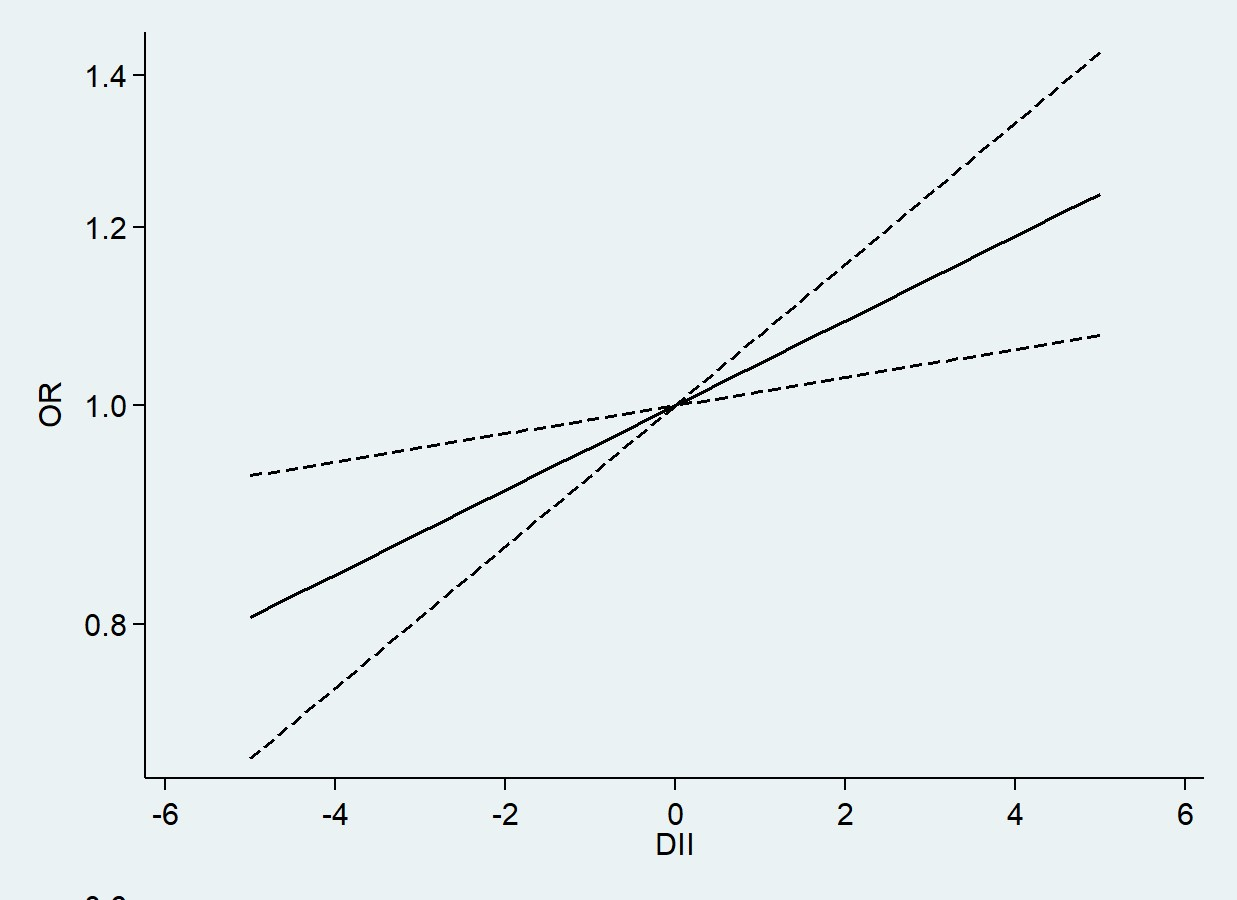


OR= 1.044 P=0.003

**Figure S13.** Linear dose–response associations between the dietary inflammatory index and metabolic syndrome in cross-sectional studies.

̶ ̶ Odds ratio; ̶̶̶̶̶ ̶̶̶̶̶ 95% confidence interval.

OR= 1.008 P=0.507

**Figure S14.** Linear dose–response associations between the dietary inflammatory index and abdominal obesity in cross-sectional studies.

̶ ̶ ̶ Odds ratio; ̶̶̶̶̶ ̶̶̶̶̶ 95% confidence interval.

OR= 0.98 P=0.548

**Figure S15.** Linear dose–response associations between the dietary inflammatory index and reduced HDL-c in cross-sectional studies.

̶ ̶ Odds ratio; ̶̶̶̶̶ ̶̶̶̶̶ 95% confidence interval.

OR= 1.019 P=0.05

**Figure S16.** Linear dose–response associations between the dietary inflammatory index and hypertriglyceridemia in cross-sectional studies.

̶ ̶ Odds ratio; ̶̶̶̶̶ ̶̶̶̶̶ 95% confidence interval.

OR= 1.11 P=0.000

**Figure S17.** Spline molel dose–response associations between the dietary inflammatory index and fasting blood sugar in cross-sectional studies.

̶ ̶ Odds ratio; ̶̶̶̶̶ ̶̶̶̶̶ 95% confidence interval.

OR= 1.015 P=0.102

**Figure S18.** Linear dose–response associations between the dietary inflammatory index and hypertension in cross-sectional studies.

̶ ̶ Odds ratio; ̶̶̶̶̶ ̶̶̶̶̶ 95% confidence interval.


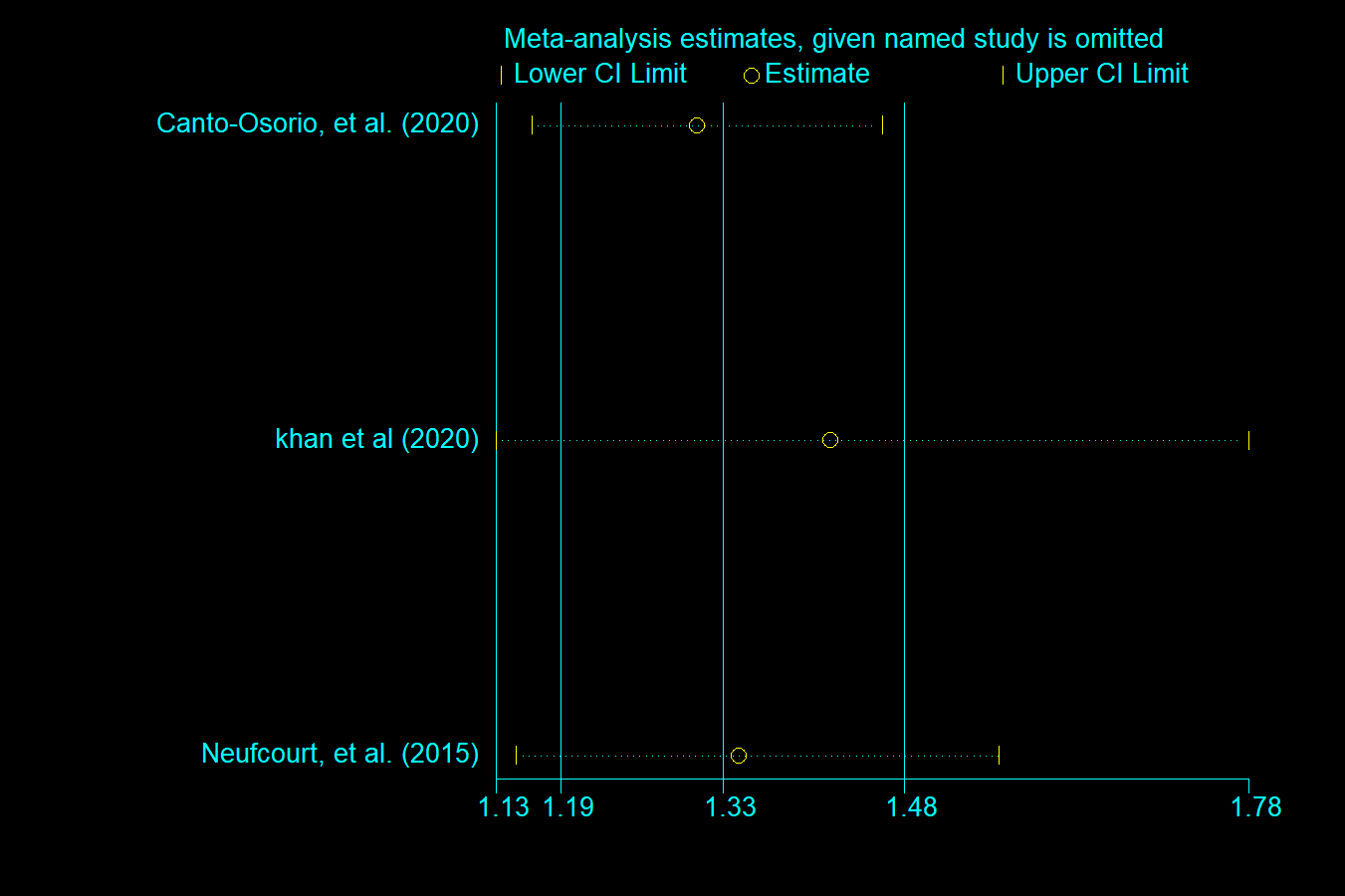


**Figure S19.** Sensitivity analyses of the overall effect size concerning the association between DII and risk of MetS in cohort studies.

*Note: the result of Sensitivity analyses was a CI range: 1.13-1.78*


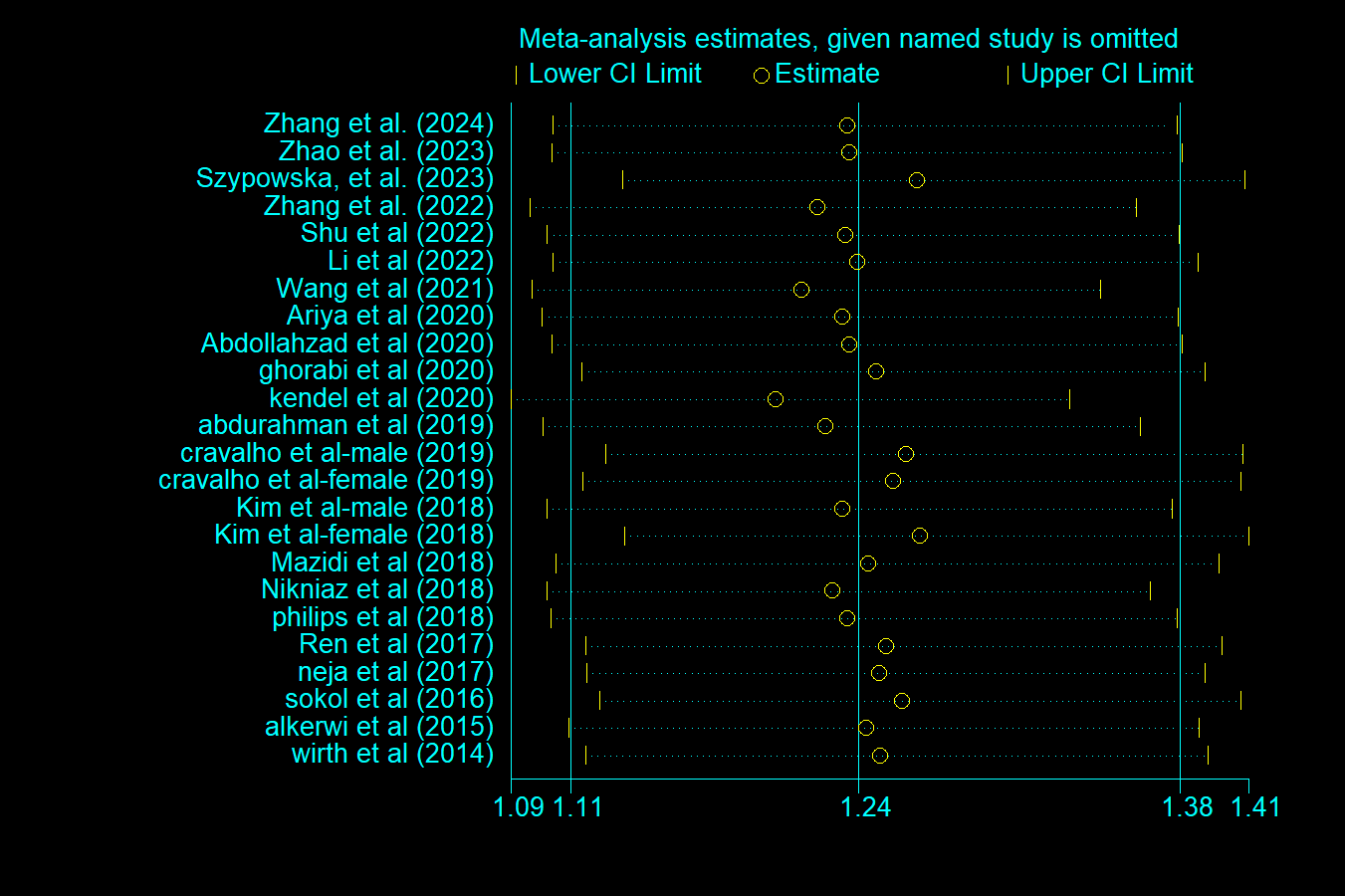


**Figure 20.** Sensitivity analyses of the overall effect size concerning the association between DII and risk of MetS in cross-sectional studies.

*Note: the result of Sensitivity analyses was a CI range: 1.09-1.41*
