# Supplementary material for: Dietary inflammatory index and its association with risk of metabolic syndrome and its components: a systematic review and Meta-analysis of Observational studies
Source: J Health Popul Nutr. 2024 Jun 19;43:87. doi: 10.1186/s41043-024-00580-w (PMC11188268; doi:10.1186/s41043-024-00580-w)
Supplement: Supplementary file 2 — Supplementary Material 2 [file 41043_2024_580_MOESM2_ESM.docx]

| **Supplemental Table 1.** Search strategy to identify observational studies reporting the associations of DII with MetS and its components | |
| --- | --- |
| **Database** | **Search terms** |
| **PubMed** | ("dietary inflammatory index"[Title/Abstract] OR "inflammatory diet*"[Title/Abstract] OR "anti-inflammatory diet*"[Title/Abstract] OR "dietary score*"[Title/Abstract] OR "pro-inflammatory diet*"[Title/Abstract] OR "inflammatory potential intake"[Title/Abstract] OR "dietary inflammatory potential score"[Title/Abstract] OR "dietary inflammatory score"[Title/Abstract] OR DII[Title/Abstract]) AND ("Metabolic syndrome*"[Title/Abstract] OR "insulin resistance syndrome"[Title/Abstract] OR "insulin resistant syndrome"[Title/Abstract] OR "syndrome x"[Title/Abstract] OR "x syndrome"[Title/Abstract] OR "metabolic cardiovascular syndrome"[Title/Abstract] OR "cardio-metabolic syndrome"[Title/Abstract] OR "glucose metabolism disorders"[Title/Abstract] OR "MetS"[Title/Abstract] OR "MetSyn" OR (("Hypertension"[Title/Abstract] OR "HP"[Title/Abstract] OR "high blood pressure") and ("Hyperlipidemia"[Title/Abstract] OR "lipid disorder")) OR (("Hypertension"[Title/Abstract] OR "HP"[Title/Abstract] OR "high blood pressure") and ("hyperglycemia"[Title/Abstract] OR "diabetes"[Title/Abstract] OR "T2DM")) OR (("Hypertension"[Title/Abstract] OR "HP"[Title/Abstract] OR "high blood pressure") and ("obesity"[Title/Abstract] OR "overweight")) OR (("Hyperlipidemia"[Title/Abstract] OR "lipid disorder") and ("hyperglycemia"[Title/Abstract] OR "diabetes"[Title/Abstract] OR "T2DM")) OR (("Hyperlipidemia"[Title/Abstract] OR "lipid disorder") and ("obesity or overweight")) OR (("hyperglycemia"[Title/Abstract] OR "diabetes"[Title/Abstract] OR "T2DM") and ("obesity"[Title/Abstract] OR "overweight"[Title/Abstract]))) |
| **Scopus** | (TITLE-ABS-KEY ("dietary inflammatory index" OR "inflammatory diet*" OR "anti-inflammatory diet*" OR "dietary score*" OR "pro-inflammatory diet*" OR "inflammatory potential intake" OR "dietary inflammatory potential score" OR "dietary inflammatory score" OR DII)) AND TITLE-ABS-KEY ("Metabolic syndrome*" OR "insulin resistance syndrome" OR "insulin resistant syndrome" OR "syndrome x" OR "x syndrome" OR "metabolic cardiovascular syndrome" OR "cardio-metabolic syndrome" OR "glucose metabolism disorders" OR "MetS" OR "MetSyn" OR (("Hypertension" OR "HP" OR "high blood pressure") and ("Hyperlipidemia" OR "lipid disorder")) OR (("Hypertension" OR "HP" OR "high blood pressure") and ("hyperglycemia" OR "diabetes" OR "T2DM")) OR (("Hypertension" OR "HP" OR "high blood pressure") and ("obesity" OR "overweight")) OR (("Hyperlipidemia" OR "lipid disorder") and ("hyperglycemia" OR "diabetes" OR "T2DM")) OR (("Hyperlipidemia" OR "lipid disorder") and ("obesity or overweight")) OR (("hyperglycemia" OR "diabetes" OR "T2DM") and ("obesity" OR "overweight")) ) |
| **Web of Science** | "dietary inflammatory index" OR "inflammatory diet*" OR "anti-inflammatory diet*" OR "dietary score*" OR "pro-inflammatory diet*" OR "inflammatory potential intake" OR "dietary inflammatory potential score" OR "dietary inflammatory score" OR DII (Topic) AND "Metabolic syndrome*" OR "insulin resistance syndrome" OR "insulin resistant syndrome" OR "syndrome x" OR "x syndrome" OR "metabolic cardiovascular syndrome" OR "cardio-metabolic syndrome" OR "glucose metabolism disorders" OR "MetS" OR "MetSyn" OR (("Hypertension" OR "HP" OR "high blood pressure") and ("Hyperlipidemia" OR "lipid disorder")) OR (("Hypertension" OR "HP" OR "high blood pressure") and ("hyperglycemia" OR "diabetes" OR "T2DM")) OR (("Hypertension" OR "HP" OR "high blood pressure") and ("obesity" OR "overweight")) OR (("Hyperlipidemia" OR "lipid disorder") and ("hyperglycemia" OR "diabetes" OR "T2DM")) OR (("Hyperlipidemia" OR "lipid disorder") and ("obesity or overweight")) OR (("hyperglycemia" OR "diabetes" OR "T2DM") and ("obesity" OR "overweight")) (Topic) |

| **Supplemental Table 2.** Detailed characteristics of studies included in the current systematic review and meta-analysis on the association between DII and MetS and its components (n=26)^1^ | | | | | | | | | | | |
| --- | --- | --- | --- | --- | --- | --- | --- | --- | --- | --- | --- |
| Author, Year  (Reference) | Country/ Type of Study | WHO region | Study period | Dietary assessment | Metabolic syndrome definition | Number of participants | Age range of participants | Female proportion | Comparison | OR/HR/RR | Adjustment |
| Zhang, et al. 2024 | China/Cross-sectional | WPR | - | FFQ | IDF | 1193 | 45.2 ± 15.0 | 26.6% | Top vs. bottom quartile | MetS: 1.38 (0.95–1.99)  AO: 1.06 (0.76–1.48)  BP: 1.22 (0.84–1.75)  HDL-C: 1.07 (0.74–1.54)  TG: 1.32 (0.95–1.82)  FBS: 1.47 (0.99–2.18) | Sex, age, and occupation |
| Zhao, et al. 2023 (19) | China/Cross-sectional | WPR | - | 24-h dietary recall | NCEP ATP III | 3843 | ≥45 | 50.90% | Top vs. bottom quartile | MetS: 1.339 (1.013-1.769)  AO: 1.205(0.806-1.803)  BP: 1.207(0.934-1.559)  HDL-C: 1.499(1.005-2.234)  TG: 1.145(0.812-1.614)  FBS: 1.432(1.095-1.873) | Age, gender, race, education, poverty–income ratio, smoking, alcohol consumption, and sedentary behavior. |
| Szypowska, et al. 2023 (20) | Poland/Cross-sectional | EUR | - | FFQ | JIS | 1570 | 35–70 | 63.70% | Top vs. bottom quartile | MetS: 0.77 (0.56-1.06)  AO: 1.22 (0.79-1.90)  BP: 0.90 (0.63-1.29)  HDL-C: 1.02 (0.71-1.47)  TG: 1.01 (0.73-1.41)  FBS: 0.78 (0.58-1.05) | Sex, age, BMI, place of residence, education level, physical activity level, and smoking. |
| Zhang, et al. 2022 (21) | China/Cross-sectional | WPR | - | FFQ | JIS | 8,180 | 20-64 | 51.4% | Top vs. bottom quartile | MetS: 1.592 (1.248, 2.030)  AO: 0.881 (0.677, 1.084) *  SBP: 0.309 (0.120, 0.498) *  DBP: 0.202 (0.058, 0.346) *  HDL-C: −0.803 (−0.986, −0.621) *  TG: 2.795 (1.003, 4.588) *  FBS: 0.229 (−0.201, 0.660) * | Gender, age, race, education level, marital status, PIR level, family history of diabetes, smoking situation, alcohol situation, physical activity |
| Shu, et al. 2022 (22) | China/Cross-sectional | WPR | - | FFQ | NCEP ATP III | 6,730 | 45–  74 | 58.78% | Top vs. bottom quartile | MetS: 1.36 (1.07-1.68)  AO: 2.14 (1.36-3.75)  BP: 1.47 (0.98-2.14)  Dyslipidaemia: 1.32 (1.14-1.67)  FBS: 1.38 (1.12-1.82) | Sex, age, residence, smoking, education, sleep duration and physical activity, BMI, energy intake |
| Li, et al. 2022 (23) | China/Cross-sectional | WPR | - | FFQ | NCEP ATP III | 1936 | ≥55 | 62% | Top vs. bottom quartile | MetS: 1.28 (1.08, 1.56)  AO: 1.59 (1.18, 2.06)  BP: 1.28 (1.09, 1.51)  HDL-C: 1.08 (0.73, 1.26)  TG: 1.39 (1.12, 1.65)  FBS: 1.23 (1.05, 1.49) | Age, sex, educational level, residence, employment status, tobacco smoking, and physical activity |
| Wang, et al. 2021 (24) | USA/Cross-sectional | AMR | - | FFQ | JIS | 276 | 36 | 100% | Top vs. bottom quartile | MetS: 4.38 (1.99, 9.63)  AO: 0.040 (0.012, 0.069) *  SBP: 0.012 (−0.003, 0.029) *  DBP: 0.035 (0.013, 0.056) *  HDL-C: −0.024 (−0.070, 0.022) *  FBS: 0.008 (−0.015, 0.031) * | Age, ethnicity, education, and household per capita income |
| Ariya, et al. 2020 (27) | Iran/Cross-sectional | EMR | - | FFQ | NCEP-ATPIII | 10,017 | 35-70 | 45.1% | Top vs. bottom quartile | MetS: 1.37 (1.18–1.59)  AO: 2.70 (2.31–3.16)  BP: 1.28 (1.11–1.47)  HDL-C: 1.29 (1.15–1.44)  TG: 0.77 (0.68–0.88)  FBS: 1.16 (1.00–1.34) | BMI and age |
| Abdollahzad, et al. 2020 (28) | Iran/Cross-sectional | EMR | - | FFQ | NCEP-ATPIII | 6,538 | 35-65 | 55.17% | Top vs. bottom quartile | MetS: 1.34 (1.01-1.77)  AO: 1.17 (0.91–1.52)  BP: 1.56 (0.97–2.50)  HDL-C: 1.24 (1.01–1.53)  TG: 1.29 (1.02–1.62)  FBS: 1.57 (1.10–2.24) | Age, sex, education, marital status, economic status, smoking, physical activity level, total energy intake, and BMI |
| Canto-Osorio, et al. 2020 (42) | Mexico/Cohort | AMR | 2004-2018 | FFQ | NCEP-ATPIII | 399 | ≥18 | 76.94% | Top vs. bottom quartile | MetS:1.99 (1.03-3.85)  AO: 2.68 (1.06, 6.79)  BP: 2.22 (1.03, 4.77)  HDL-C: 1.27 (0.31, 5.19)  TG: 2.28 (1.13, 4.57)  FBS: 1.33 (0.68, 2.58) | Age, sex, educational level, smoking status, physical activity, sleep time, and energy intake. |
| Ghorabi, et al. 2020 (26) | Iran/Cross-sectional | EMR | - | FFQ | NCEP-ATPIII | 404 | ≥18 | 37.6% | Top vs. bottom tertile | MetS: 0.92 (0.48-1.76)  AO: 1.11 (0.45, 2.71)  SBP: 0.47 (0.22, 1.93)  DBP: 0.45 (0.21, 1.10)  HDL-C: 2.71 (1.34, 5.47)  TG: 0.71 (0.38, 1.32)  FBS: 0.87 (0.48, 1.57) | Age, gender, energy intake, marital status, physical activity, education status, smoking, economic status, supplementation, and BMI. |
| Kenđel, et al. 2020 (25) | Croatia/Cross-sectional | EUR | - | FFQ | IDF | 366 | NS | 51.6% | Per DII unit | MetS: 2.31 (1.61-3.31)  AO: 1.32 (0.94–1.85)  BP: 1.28 (1.01–1.64)  HDL-C: 1.27 (0.67–2.43)  TG: 1.29 (0.98–1.69)  FBS: 0.85 (0.66–1.10) | Sex, age, educational level, smoking, physical activity, body mass index, and energy intake |
| Khan, et al. 2020 (41) | Korea/Cohort | SEAR | 2004-2013 | FFQ | NCEP-ATPIII | 157,812 | 40–79 | 66.22% | Top vs. bottom quintile | MetS: 1.31 (1.15-1.49)  AO: 1.37 (1.24-1.51)  BP: 1.24 (1.15-1.34)  HDL-C: 1.63 (1.44-1.84)  TG: 1.24 (1.14-1.35)  FBS: 1.18 (1.09-1.26) | Sex, age, smoking, alcohol drinking, physical activity, BMI, family history of diabetes mellitus, family history of hypertension, and energy intake. |
| Abdurahman, et al. 2019 (30) | Iran/Cross-sectional | EMR | - | FFQ | NCEP-ATPIII | 300 | 43.4 | 84.7% | Top vs. bottom quintile | MetS: 2.58 (1.19–5.59)  AO: 0.58 (0.16–2.05)  BP: 1.66 (0.83–3.34)  HDL-C: 1.19 (0.55–2.57)  TG: 1.66 (0.82–3.37)  FBS: 1.89 (0.92–3.91) | Age, sex, physical activity level, body mass index, and history of chronic diseases |
| Carvalho, et al. 2019 (28) | Brazil/Cross-sectional | AMR | - | FFQ | JIS | 2017 | 23-25 | 53.1% | Per DII unit | Male: 0.98 (0.91-1.07)  Female: 1.05 (0.91-1.2) | Age, family income in multiples of the month minimum wage, and schooling. |
| Kim, et al. 2018 (35) | Korea/Cross-sectional | SEAR | - | 24-h dietary recall | NCEP-ATPIII and the obesity guidelines of the Obesity Society of Korea | 9291 | 19–65 | 60.3% | Top vs. bottom quartile | Male  MetS: 1.40 (1.06-1.85)  AO: 1.07 (0.72–1.61)  BP: 1.14 (0.88–1.46)  HDL-C: 0.93 (0.72–1.21)  TG: 1.22 (0.97–1.53)  FBS: 1.30(1.02–1.65)  Female  MetS: 0.83 (0.66-1.04)  AO: 1.35 (0.94–1.94)  BP: 1.10 (0.87–1.38)  HDL-C: 0.85 (0.71–1.04)  TG: 1.07 (0.84–1.38)  FBS: 0.95 (0.77–1.18) | Age, BMI, education, alcohol consumption, smoking, physical activity, and total calorie intake |
| Mazidi, et al. 2018 (34) | United States/Cross-sectional | AMR | - | NS | NCEP-ATPIII | 17,689 | ≥18 | 51.70% | Top vs. bottom quartile | MetS: 1.23 (1.07-1.41)  AO: 1.28 (1.17-1.52)  BP: 1.21 (1.02-1.43) | Age, sex, race, smoking, energy intake, education level, marital status, and body mass index |
| Nikniaz, et al. 2018 (33) | Iran/Cross-sectional | EMR | - | FFQ | NCEP-ATPIII | 606 | 18-64 | 54.50% | Top vs. bottom quartile | MetS: 2.26 (1.03-4.92)  AO: 0.86 (0.39, 1.91)  BP: 1.18 (0.47, 2.96)  HDL-C: 0.83 (0.44, 1.55)  TG: 1.31 (0.66, 2.58)  FBS: 2.56 (1.01, 7.05) | Smoking status (smoker and non-smoker), physical activity (low, moderate, high), sex, age, and BMI. |
| Phillips, et al. 2018 (32) | Ireland/Cross-sectional | EUR | - | FFQ | NCEP-ATPIII | 1992 | 50-69 | 51% | Top vs. bottom half | MetS: 1.37 (1.01-1.88) | Age, gender, BMI, physical activity, smoking status, alcohol consumption, and use of anti-inflammatory and lipid-lowering medication. |
| Ren, et al. 2018 (31) | China/Cross-sectional | WPR | - | 24-h dietary recall | JIS | 1712 | 18–75 | 66% | Top vs. bottom tertile | MetS: 1.02 (0.75-1.4)  AO: 0.86 (0.59–1.24)  BP: 1.40 (1.03–1.89)  HDL-C: 1.17 (0.88–1.56)  TG: 1.03 (0.78–1.37)  FBS: 0.85 (0.64–1.14) | Age, gender, city, education level, family monthly expenditure on food, smoking status, and BMI. |
| Naja, et al. 2017 (36) | Lebanon/Cross-sectional | EMR | - | FFQ | IDF | 331 | ≥18 | 51.06% | Top vs. bottom quintile | MetS: 0.72 (0.31-1.67)  AO: 0.66 (0.29–1.48)  BP: 0.40 (0.23–1.04)  HDL-C: 0.74 (0.31–1.75)  TG: 0.84 (0.35–1.03)  FBS: 1.80 (0.80–4.01) | Age, sex, marital status, education, crowding index, physical activity, and smoking. |
| Sokol, et al. 2016 (37) | Poland/Cross-sectional | EUR | - | FFQ | Other* | 3862 | 45-64 | 66.60% | Top vs. bottom quartile | MetS: 0.96 (0.77-1.19)  AO: 0.79 (0.61–1.03)  BP: 1.05 (0.86–1.28)  HDL-C: 0.62 (0.48–0.80)  TG: 1.04 (0.84–1.30)  FBS: 1.11 (0.91–1.34) | BMI and age |
| Neufcourt, et al. 2015 (44) | France/Cohort | EUR | 1994-2002 and 2007-2009 | Food records | JIS | 3726 | 35-60 | 63.50% | Top vs. bottom quartile | MetS: 1.39 (1.01-1.92)  AO: 86.60 (86.18-87.03) ^†^  SBP: 126.1 (125.1-127.1) ^†^  DBP: 77.74 (77.06-78.42) ^†^  HDL-C: 1.50 (1.48-1.52) ^†^  TG: 1.05 (1.03-1.07) ^†^  FBS: 5.25 (5.22-5.28) ^†^ | Age, gender, supplementation group, energy intake, education level, smoking status, physical activity, and BMI |
| Alkerwi, et al. 2014 (39) | Luxembourg/Cross-sectional | EUR | - | FFQ | NCEP-ATPIII | 1352 | 18-69 | 51.40% | Top vs. bottom half | MetS: 1.18 (0.81-1.71)  AO: 1.12 (0.81-1.56)  BP: 0.85 (0.61-1.18)  HDL-C: 1.46 (1.00-2.13)  TG: 1.17 (0.82-1.67)  FBS: 1.30 (0.90-1.89) | Age, sex, education, income smoking status, and physical activity |
| Wirth, et al. 2014 (38) | United States/Cross-sectional | AMR | - | FFQ | NCEP-ATPIII | 447 | NS | 25.0% | Top vs. bottom quartile | MetS: 0.87 (0.46-1.63)  AO: 0.93 (0.52–1.67)  BP: 1.14 (0.64–2.02)  HDL-C: 1.03 (0.59–1.83)  TG: 0.77 (0.42–1.42)  FBS: 2.03 (1.08–3.82) | Age and sex |

^1^Values are OR/HR/RR unless otherwise indicated. *β (95% confidence interval); ^●^β (Standard Error); ^†^means (95% confidence interval)

Abbreviations: AO: Abdominal obesity, DII: Dietary Inflammatory Index; EMR, Eastern Mediterranean Region; AMR, Americas Region; EUR, European Region; SEAR, South-East Asia Region; WPR, Western Pacific Region; NS, Not Specified; FFQ, Food Frequency Questionnaire; NCEP-ATPIII, The Third Report of the National Cholesterol Education Program Expert Panel on Detection, Evaluation, and Treatment of High Blood Cholesterol in Adults (Adult Treatment Panel III); IDF, The International Diabetes Federation Criteria; JIS, Joint Interim Statement; MetS: Metabolic syndrome.

| **Supplemental Table 3**. Quality assessment of the studies included in the current systematic review and meta-analysis of the association between DII and the MetS and its components using the ROBINS-I tool^1^ | | | | | | | | |
| --- | --- | --- | --- | --- | --- | --- | --- | --- |
|  | Bias due to confounding | Bias due to the selection of participants | Bias due to exposure assessment | Bias due to misclassification during follow-up | Bias due to  missing data | Bias due to  measurement of the outcome | Bias due to selective  reporting of the results | Overall  judgment |
| Zhang, et al. 2024 | Moderate | Moderate | Low | Low | Low | Low | Low | Moderate risk of bias |
| Zhao, et al. 2023 | Moderate | Low | Low | Low | Low | Low | Low | Moderate risk of bias |
| Szypowska, et al. 2023 | Moderate | Moderate | Low | Low | Low | Low | Low | Moderate risk of bias |
| Zhang, et al. 2022 | Moderate | Low | Low | Low | Low | Low | Low | Moderate risk of bias |
| Shu, et al. 2022 | Low | Moderate | Low | Low | Low | Low | Low | Moderate risk of bias |
| Li, et al. 2022 | Moderate | Moderate | Low | Low | Low | Low | Low | Moderate risk of bias |
| Wang, et al. 2021 | Moderate | Moderate | Low | Low | Low | Low | Low | Moderate risk of bias |
| Ariya, et al. 2020 | Moderate | Moderate | Low | Low | Low | Low | Low | Moderate risk of bias |
| Abdollahzad, et al. 2020 | Low | Moderate | Low | Low | Low | Low | Low | Moderate risk of bias |
| Canto-Osorio, et al. 2020 | Low | Low | Low | Low | Low | Low | Low | Low risk of bias |
| Ghorabi, et al. 2020 | Low | Moderate | Low | Low | Low | Low | Low | Moderate risk of bias |
| Kendel, et al. 2020 | Low | Moderate | Low | Low | Low | Low | Low | Moderate risk of bias |
| Khan, et al. 2020 | Low | Low | Low | Low | Low | Low | Low | Low risk of bias |
| Abdurahma, et al. 2019 | Moderate | Moderate | Low | Low | Low | Low | Low | Moderate risk of bias |
| Carvalho, et al. 2019 | Moderate | Moderate | Low | Low | Low | Low | Low | Moderate risk of bias |
| Kim, et al. 2018 | Low | Moderate | Low | Low | Low | Low | Low | Moderate risk of bias |
| Mazidi, et al. 2018 | Low | Moderate | Low | Low | Low | Low | Low | Moderate risk of bias |
| Nikniaz, et al. 2018 | Moderate | Moderate | Low | Low | Low | Low | Low | Moderate risk of bias |
| Phillips, et al. 2018 | Moderate | Moderate | Low | Low | Low | Low | Low | Moderate risk of bias |
| Ren, et al. 2018 | Moderate | Moderate | Low | Low | Low | Low | Low | Moderate risk of bias |
| Naja, et al. 2017 | Moderate | Moderate | Low | Low | Low | Low | Low | Moderate risk of bias |
| Sokol, et al. 2016 | Moderate | Moderate | Low | Low | Low | Low | Low | Moderate risk of bias |
| Neufcourt, et al. 2015 | Low | Low | Low | Low | Low | Low | Low | Low risk of bias |
| Alkerwi, et al. 2014 | Moderate | Moderate | Low | Low | Low | Low | Low | Moderate risk of bias |
| Wirth, et al. 2014 | Moderate | Moderate | Low | Low | Low | Low | Low | Moderate risk of bias |

^1^DII: Dietary Inflammatory Index; MetS: Metabolic syndrome; ROBINS-I: Risk Of Bias In Non-randomized Studies-of Interventions.
